# Suppression of exciton–vibration coupling via modulated insulator dilution for thickness-tolerant organic solar cells

**DOI:** 10.1093/nsr/nwaf387

**Published:** 2025-09-16

**Authors:** Zhen Fu, Jia-Wei Qiao, Kang-Ning Zhang, Wen-Qing Zhang, Ming-Xu Zhou, Jin-Qun Xu, Peng Lu, Hang Yin, Xiao-Yan Du, Xiao-Tao Hao

**Affiliations:** School of Physics, State Key Laboratory of Crystal Materials, Shandong University, Jinan 250100, China; School of Physics, State Key Laboratory of Crystal Materials, Shandong University, Jinan 250100, China; School of Physics, State Key Laboratory of Crystal Materials, Shandong University, Jinan 250100, China; School of Physics, State Key Laboratory of Crystal Materials, Shandong University, Jinan 250100, China; School of Physics, State Key Laboratory of Crystal Materials, Shandong University, Jinan 250100, China; School of Physics, State Key Laboratory of Crystal Materials, Shandong University, Jinan 250100, China; School of Physics, State Key Laboratory of Crystal Materials, Shandong University, Jinan 250100, China; School of Physics, National Demonstration Center for Experimental Physics Education, Shandong University, Jinan 250100, China; School of Physics, State Key Laboratory of Crystal Materials, Shandong University, Jinan 250100, China; School of Physics, State Key Laboratory of Crystal Materials, Shandong University, Jinan 250100, China; School of Physics, State Key Laboratory of Crystal Materials, Shandong University, Jinan 250100, China; School of Chemistry, The University of Melbourne, Parkville 3010, Australia

**Keywords:** organic photovoltaic, thickness-tolerant, exciton–vibration coupling

## Abstract

Developing thickness-tolerant organic solar cells (OSCs) is imperative for scalable roll-to-roll fabrication, yet prevalent systems suffer from severe efficiency losses at increased active-layer thicknesses. Strong exciton–vibration coupling critically impedes exciton transport and triggers detrimental non-radiative recombination in thick films, curtailing exciton diffusion length. Herein, we propose a modulated insulator dilution (MID) strategy, employing tailored molecular weight polypropylene (PP), to achieve high-performance OSCs with exceptional thickness tolerance. Driven by a polymer swelling effect during processing, PP incorporation orders acceptor molecular packing, optimizing fibrous nanomorphology and enhancing π–π interactions within PM6:L8-BO systems. This MID approach effectively suppresses exciton–vibration coupling and optimizes excited-state dynamics. By localized-to-delocalized exciton diffusion enhancement, PP incorporation boosts the efficiency of a 500 nm device to 15.92%, which is one of the highest values among 500 nm OSCs. Efficiency remains substantial (∼12%) even at a challenging 1-μm thickness. This work underscores molecular weight-modulated insulator dilution as a pivotal strategy for exciton management and performance enhancement, providing a crucial pathway toward the industrialization of organic photovoltaics.

## Teaser

This work introduces a novel strategy of modulated insulator dilution (MID) to suppress exciton–vibration coupling, improves the excited state evolution process and ultimately achieves exceptional thickness tolerance in OSCs.

## INTRODUCTION

Solution-processed organic solar cells (OSCs) with bulk heterojunction (BHJ) architecture have received intense study and experienced dramatic development owing to their low cost, lightweight nature and flexibility [1–3]. However, the majority of these devices exhibit optimal performance when the layer thickness is maintained at approximately 100 nm, which decreases drastically with the increase of the active-layer thickness, limiting their application in roll-to-roll large-scale solution printing technology [4,5]. Increasing the active-layer thickness enhances photon absorption and facilitates diffusion of more excitons to the donor/acceptor (D/A) interfaces, where they can dissociate into charge carriers [6,7]. Consequently, the diffusion length (*L_D_*) of excitons plays a critical role in determining the quantity of excitons that reach the D/A interfaces [8,9]. Strong exciton–vibration coupling can hinder exciton transport and induce undesirable non-radiative recombination, resulting in a shortened exciton *L_D_* and constrained exciton dissociation in OSCs [10,11]. As a result, suppressing exciton–vibration coupling is crucially important for achieving highly thickness-tolerant OSCs.

Recent reports have begun to focus on the significant role of exciton–vibration coupling in carrier dynamics and device performance [12,13]. Huang *et al.* enhanced molecular rigidity, suppressed structural relaxation and weakened exciton–vibration coupling strength by tuning intramolecular non-covalent interactions [12]. Zhao *et al.* propose an easy method to efficiently reduce vibrational frequency of the molecular skeleton and suppress exciton–vibration coupling to decrease the non-radiative decay rate and thus enable high-efficiency OSCs [14]. Meanwhile, extensive research has focused on enhancing thick-film-tolerant OSCs by developing novel photovoltaic materials with long exciton *L_D_*, and optimizing the nanoscale morphology of the active layer to improve exciton diffusion [8,15,16]. One strategy is blending insulating polymers with organic semiconductors, which has shown promise in extended exciton *L_D_* in highly thickness-tolerant OSCs [17,18]. The critical influence of insulating polymer molecular weight and exciton–vibration coupling on device performance across varying thicknesses is crucially important. However, the exciton–vibration coupling variation and unique mechanisms of insulators with diverse molecular weights under various active-layer thicknesses are still ambiguous. This represents a significant gap in understanding of the fundamental mechanisms that govern charge transport and exciton dynamics in these systems.

In this study, we employed a novel strategy of modulated insulator dilution (MID) to suppress exciton–vibration coupling, reduce non-radiative recombination, extend exciton *L_D_* and ultimately achieve highly thickness-tolerant OSCs. Specifically, we introduced polypropylene (PP) with varying molecular weights into PM6:L8-BO systems of different thicknesses. The introduction of PP with various molecular weights effectively promotes the ordered stacking of acceptor molecules, enhances the interaction between small molecules, optimizes the size of L8-BO fibers and facilitates the transport of more excitons within the acceptor phase, thereby achieving localized-to-delocalized exciton diffusion enhancement. Consequently, we observed that medium-molecular-weight PP can boost the efficiency of a 500 nm thick device to 15.92%, which is one of the highest values among 500 nm OSCs, and even when the film thickness reaches 1 μm, the efficiency of the device can still be maintained at approximately 12%. These results highlight the significant impact of insulator molecular weight on exciton diffusion and overall device performance. Our findings provide valuable criteria for selecting insulator molecular weights to enhance exciton diffusion, and offer insights into the design of highly thickness-tolerant OSCs.

## RESULTS AND DISCUSSION

**Figure 1.**
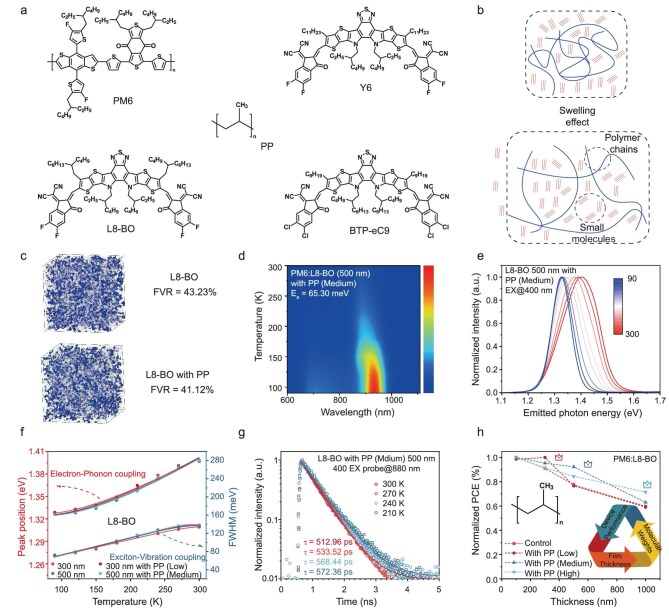
(a) Chemical structures of different materials. (b) Schematic illustration of swelling effect. (c) Representative snapshots of molecular dynamics (MD) simulated molecular packing morphologies. (d) Temperature-dependent PL spectra of 500 nm PM6:L8-BO with PP (medium). (e) Temperature-dependent PL spectra of 500 nm L8-BO with PP (medium). (f) Peak location and FWHM of the emission peak as a function of temperature in respective films. (g) Temperature-dependent TRPL profiles of blends (pump at 800 nm). (h) The variation trend of PCE in different systems increases with the active-layer thickness.

Figure 1a shows the chemical structures of the donors, acceptors and insulators with different molecular weights (low, medium and high; the specific molecular weight values are detailed in the Supplementary data) used in this study. As shown in Figs S1–S5 and Tables S1–S3, among the commonly used non-fullerene acceptors, Y6 shows the best thickness tolerance, followed by L8-BO and BTP-eC9. On this basis, we chose PP with varying molecular weights to modulate non-fullerene systems with different thicknesses, thereby achieving devices with high thickness tolerance. Representative height profiles are presented in Fig. S6. The Flory–Huggins interaction parameter χ and the wetting coefficient ω were computed based on contact angle measurements to elucidate the miscibility and phase distribution of the materials (Fig. S7 and Table S4) [19]. The obtained χ values for PP (low)/PM6 (300 nm), PP (low)/L8-BO (300 nm), PP (medium)/PM6 (500 nm) and PP (medium)/L8-BO (500 nm) were found to be 0.54, 0.08, 3.57 and 0.50, respectively, indicating a greater miscibility between PP and L8-BO. Driven by the swelling effect of polymers, the insulating polymer PP tends to swell during the solution process [20] (Fig. 1b). The diffusion rate of L8-BO exceeds that of PP, enabling it to infiltrate the interior of PP and induce volumetric expansion of PP. This expansion subsequently decreases the distance between L8-BO molecules, leading to denser packing and enhanced π–π interactions among them. The improved packing and intensified interactions of L8-BO facilitate the formation of more orderly molecular arrangements and higher crystallinity, which are crucial for reducing exciton–vibration coupling and boosting exciton diffusion.

In order to explore the effect of PP swelling, the free volume ratios (FVRs) of different systems have been investigated by atomistic molecular dynamics simulations (for details see Supplementary data). As shown in Fig. 1c, the L8-BO system possesses an FVR of 43.23%, and the L8-BO with PP system allows decreased FVR values of 41.12%, respectively. The reduced FVR allows less space for molecular motion, leading to a suppressed non-radiative decay process [21]. Our results also indicate that in thick-film conditions (where L8-BO molecules are abundant), higher-molecular-weight PP more effectively reduces the free volume ratio due to its superior space-filling capacity (Fig. S8). To further investigate the physical basis of the swelling effect driving L8-BO packing, we calculated the cohesive energy density (CED) of L8-BO, and found that incorporating PP increases the CED by enhancing intermolecular van der Waals interactions (Fig. S9). This increase in CED directly correlates with improved π–π stacking, as evidenced by grazing-incidence wide-angle X-ray scattering (GIWAXS), and extended exciton delocalization observed in TA spectroscopy, which will be detailed in the subsequent discussion. These findings demonstrate that PP modulates L8-BO packing through swelling-induced intermolecular interaction strengthening [7].

To obtain the specific information about molecular vibration, Raman spectroscopy was employed to acquire specific molecular vibrational information from L8-BO films of different thicknesses blended with PP of corresponding molecular weights. As shown in Fig. S10, the main Raman peaks at 1447 cm^−1^ represent the C=C vibration of the fused thiophenes, and 1547 cm^−1^ is the alkene peak [11,22]. The peaks at 1290 cm^−1^ correspond to the core benzodithiophene (BTT) unit [23]. The sharp band with a narrow full width at half-maximum (FWHM) at the 1447–1547 cm^−1^ region for thick films with PP indicate the enhanced molecular order and packing. The 1290 cm⁻^1^ peak exhibits a clear redshift to a lower wavenumber in the blend with PP. This shift is a classic signature of strengthened intermolecular π–π interactions, which effectively suppresses its vibrational frequency.

Figure S11 illustrates the normalized absorption spectra of PM6:L8-BO in both control samples and those incorporating PP followed by thermal annealing. The absorption peak of L8-BO films modulated by insulator dilution with various molecular weights exhibited varying degrees of redshift compared to the control films. Specifically, the absorption peak of the optimized 500 nm film shifted to red by ∼13 nm (from 789.3 to 801.9 nm), suggesting the formation of J-aggregates by L8-BO molecules in the films [24]. A similar pattern of J-aggregation was observed in the 300 nm PM6:L8-BO films with PP (low). To evaluate the effect of changes in insulator dilution with various molecular weights on exciton–vibration coupling, we present the temperature-dependent photoluminescence (PL) spectra with 400 nm excitation in Fig. 1d and Fig. S12. Using the measured temperature dependence of the PL emission spectra, the exciton binding energy (${E}_a$) was obtained by fitting the curves using Equation (1):


(1) \begin{eqnarray*}
{I}_T = \frac{{{I}_0}}{{1 + A{e}^{ - {E}_a/{k}_BT}}},
\end{eqnarray*}

where *I_T_, e, k_B_* and *T* represent the integrated PL intensity, natural constant, Boltzmann constant and temperature, respectively. *I_0_* and *A* are the fitting parameters [25]. The calculated *E_a_* of the PM6:L8-BO film with different thicknesses significantly decreased after blending with PP, explaining the efficient exciton dissociation process in the active layer, thus promoting the generation of free electrons, achieving improved device thickness tolerance. The temperature-dependent PL spectra of L8-BO were systematically measured with a redshift trend observed in the emission peak positions, indicating renormalization of band energies [26]. (Fig. 1e and Fig. S13) The temperature dependency of the peak energies, extracted from the PL spectra, is depicted in Fig. 1f. Quantitative analyses are detailed in Table S5. The total FWHM of each emission peak can be described as the sum of the three terms using Equation (2):


(2) \begin{eqnarray*}
{\mathrm{\Gamma }}\!\left( T \right) = {\Gamma }_i + \alpha \exp \left( {\frac{{ - {E}_A}}{{{k}_BT}}} \right) + \beta T,
\end{eqnarray*}

where *Γ_i_* is the inhomogeneous line width of the semiconductor, caused by variations in composition, morphology, size and other factors [27]. The calculated activation energy *E*_A_ in L8-BO-based 500 nm film decreased from 245.32 to 203.79 meV after blending with PP (medium), indicating a faster interfacial exciton dissociation process during hole transfer (HT) kinetics. Moreover, the exciton–vibration coupling coefficient (*β*) of L8-BO is significantly reduced under an MID strategy compared to pristine films. This decrease in *β* suggests that the blending with PP effectively suppresses the exciton–vibration coupling and improves exciton diffusion. To verify reduced electron–phonon coupling exclusively attributable to PP, we conducted GIWAXS measurements to explore L8-BO crystallization characteristics with different thicknesses (Fig. S14 and Table S6). Our findings reveal that increasing the thickness of the L8-BO layer from 300 to 500 nm results in an increase of the crystalline coherence length (CCL) from 14.411 to 14.702 Å. However, we also observed a rise in the *β* parameter from 380.90 to 388.47 μeV K^−1^ as the thickness increased. This observation suggests that thicker films tend to additional lattice defects, which in turn enhance exciton–vibration coupling. In contrast, when an appropriately matched molecular-weight PP is incorporated at the same thickness, the CCL increases while *β* decreases. This finding underscores that PP not only refines molecular packing but also suppresses lattice defect-mediated coupling, thereby mitigating the thickness-induced increase in exciton–vibration coupling. The temperature-dependent emission peak energy can be described by Equation (3):


(3) \begin{eqnarray*}
E\!\left( T \right) = E\!\left( {\mathrm{0}} \right) - \frac{{2a}}{{\exp \left( {{{\theta } / {T}} - 1} \right)}},
\end{eqnarray*}

where *E*(0) is the bandgap energy at 0 K, *a* is the strength of the electron–phonon interaction (both acoustic and optical phonons) and *θ* is the average phonon temperature [28]. Upon fitting Equation (3), we found that the strength of the electron–phonon interactions in L8-BO (300 nm) with PP (low) and L8-BO (500 nm) with PP (medium) were diminished relative to those in pristine L8-BO.

To determine the molecular motion before and after insulator molecular-weight modulation, temperature-dependent time-resolved PL (TRPL) measurements were also performed on different films (Fig. 1g and Fig. S15). The instrument response function is shown in Fig. S16. The time of the instrument response function can be obtained by fitting and was 26.5 ps. This response time is significantly shorter than the exciton lifetimes we have measured, which allows us to confidently state that our data are accurate and not compromised by the limitations of our equipment. Compared with neat L8-BO films with different thicknesses, adding different molecular-weight PP leads to a reduced temperature dependence under 800 nm excitation. These observations illustrate the suppression of the vibrations and torsion in the L8-BO molecules [13].

As illustrated in Fig. 1h, the MID with various molecular weights is demonstrated to significantly enhance the efficiency of thick-film devices across a broad range of thicknesses. This approach effectively enables directional control of the performance as a function of thickness in thick-film devices, thereby providing a rational strategy to optimize device performance and improve thickness tolerance. As the film thickness increases, the number of L8-BO molecules also rises. During the swelling process, polymers with different molecular weights accommodate varying numbers of small molecules [29]. Low-molecular-weight PP cannot sufficiently accommodate the L8-BO through the swelling effect. The high-molecular-weight PP can adapt to the ingress of more L8-BO through segmental motion and extension. This enables the effective formation of a swelling effect and aggregation of L8-BO molecules, even in thick-film active layers.

**Figure 2.**
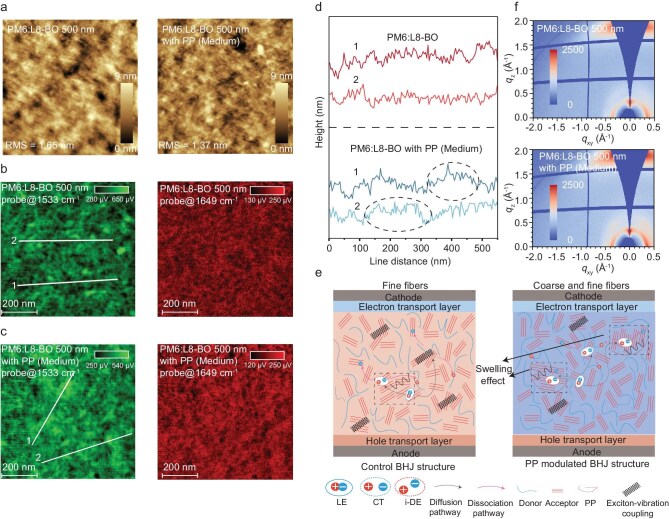
(a) AFM height images of different systems. (b and c) PiFM images at a wavenumber of 1533 cm^−1^ (representing L8-BO) and 1649 cm^−1^ (representing PM6). (d) The line profile of nanofiber cross-sections obtained by PiFM map. (e) Schematic illustration of the phase separation. (f) 2D GIWAXS patterns.

To investigate the morphological evolution of the films with different thicknesses, we conducted various morphological characterization methods. Atomic force microscopy (AFM) height images are shown in Fig. 2a and Fig. S17. The 300 nm film exhibits a distinct fiber-like morphology, while the uniformity of the film decreases with increasing thickness. The introduction of corresponding molecular weight PP promotes the improvement of film morphology, presenting a significant increase in surface uniformity [30].

However, AFM images do not distinguish between donor and acceptor morphology, and therefore cannot selectively map the fibrous nanostructure formed by different components. To address this challenge, we resorted to the photo-induced force microscopy (PiFM) technique, which was demonstrated to selectively image either donor or acceptor nanostructures through the analyses of stretching of feature bonds [30,31] (Fig. 2b–d and Figs S18–S20). We performed Fourier-transform infrared (FT-IR) spectra of neat PM6 and L8-BO films to detect their characteristic peak positions. As shown in Fig. S21, PM6 shows a unique peak at a wavenumber of 1649 cm^−1^ from alkene vibrations, L8-BO shows a unique peak at a wavenumber 1533 cm^−1^ from N–H stretching; PP does not exhibit characteristic peaks in the range of 1500–1700 cm⁻¹ [30,32]. The absorption at these different wavenumbers provides a window for us to use PiFM to distinguish the crystal structure of the donor and acceptor in the active layer at the nanometer level. On the one hand, a higher number of D/A interfaces can promote exciton generation, corresponding to a denser and more refined nanoscale fiber network. However, an excess of D/A interfaces can lead to exciton annihilation. On the other hand, effective charge transport after exciton dissociation needs large, pure donor and acceptor domains to create transport channels, which corresponds to a nanoscale fiber network with strong crystallinity. However, excessively large phase separation can also lead to severe exciton recombination [33]. After adding PP of corresponding molecular weight into the blend, the fiber network of the acceptor phase exhibits a coexistence of coarse and fine fibers. Fine fibers originate from the original aggregation process of L8-BO, while coarse fibers originate from the swelling effect brought about by the introduction of PP, which promotes more L8-BO aggregation, forms a more ordered molecular arrangement and increases the size of L8-BO fibers. As shown in Fig. 2e, the formation of a multiscale nanoscale interpenetrating fiber network structure helps the achievement of efficient exciton dissociation while suppressing exciton recombination processes and prolonging exciton diffusion. Amorphous polymers such as polyvinyl alcohol (PVA) have loosely packed molecular chains with relatively weak intermolecular forces. In contrast to amorphous polymers, PP possesses a certain degree of molecular chain regularity, featuring both crystalline and amorphous regions [34]. This semi-crystalline architecture enables localized chain expansion, creating transient free-volume regions that accommodate small molecules without disrupting long-range order. As shown in Fig. S22, binding-energy calculations performed with Gaussian further reveal that PP interacts more strongly with L8-BO (−30.21 kcal mol⁻^1^) than with PM6 (−26.72 kcal mol⁻^1^), confirming preferential PP–acceptor contacts. Consequently, PP acts as a transient and swollen scaffold that guides L8-BO self-aggregation, yielding films with larger coherence lengths and enhanced phase separation.

The GIWAXS measurement was used to study the molecular packing and crystallinity in different thickness films [31]. Figure 2f and Fig. S23 show the 2D-GIWAXS images and the corresponding line profiles of different blended films. For these blend films, (100) peaks are observed in the in-plane direction and (010) peaks are found in the out-of-plane direction, which suggests that all the blend films show a predominantly face-on orientation. Integration along the polar angle direction suggests that MID significantly enhances the face-on microcrystal ratio of acceptor molecules in different thickness films (Fig. S24). In addition, the CCL, which is closely related to the charge transport and device performance, is extracted from GIWAXS line profiles according to the Scherrer equation to quantify the molecular crystallinity, as summarized in Table S7. In the 300 and 500 nm PM6:L8-BO blending with corresponding molecular weight PP, the CCL values associated with π–π stacking (010) are 28.765 and 29.182 Å, respectively, greater than the control 300 and 500 nm blends (26.597 and 28.497 Å, respectively). The elongated CCLs indicate the increased crystallinity and more orderly stacking of molecules. The well-developed crystallinity is beneficial for the charge transport and therefore results in a higher device thickness tolerance. As mentioned above, we conducted GIWAXS measurements on 300 and 500 nm L8-BO films both before and after blending with PP of matched molecular weights. For the 300 nm L8-BO layer, incorporating PP (low) increases the CCL from 14.411 to 19.787 Å while simultaneously reducing the d-spacing from 3.38 to 3.36 Å. In the 500 nm layer, blending with PP (medium) yields an even more pronounced effect: the CCL rises from 14.702 to 23.155 Å and the d-spacing decreases from 3.38 to 3.34 Å. These consistent trends—longer CCL and shorter d-spacing—clearly demonstrate that PP promotes denser molecular packing and superior ordering of L8-BO.

**Figure 3.**
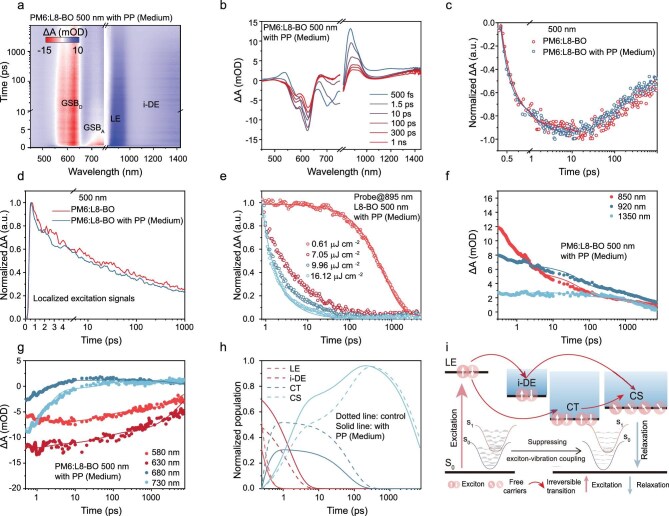
The 2D TA profile (a) and TA spectra (b) at different time delays pumped at 800 nm. (c) Dynamics of the rising signal of PM6 GSB in different films. (d) TA dynamics of the LE signals in different films. (e) Pump-fluence-dependent TA kinetics of L8-BO with PP (medium). (f–h) Global fitting analysis of TA data recorded from PM6:L8-BO with PP (medium) film. (i) Schematics of i-DE-mediated and CT-mediated charge generation progress.

We performed transient absorption (TA) spectroscopy to gain a deeper insight into the dynamics of charge generation and recombination in a variety of thick films. The 2D color plots of the TA spectra for these systems are illustrated in Fig. 3a and Fig. S25, and the corresponding TA spectra recorded at different time delays are presented in Fig. 3b and Fig. S26. A pump pulse wavelength of 800 nm was used to selectively excite L8-BO. Both blends exhibited ground state bleaching (GSB) of L8-BO around 750 nm, localized excitation (LE) at 900 nm, intra-delocalized exciton (i-DE) between 1300 and 1500 nm, and GSB of PM6 between 550 and 630 nm, consistent with previous findings, and attests to the presence of HT kinetics [22]. The TA data obtained from the 500 nm blend with PP (medium) under excitation at 800 nm revealed that the HT from the acceptor to the polymer donor-initiated charge generation. We applied biexponential fitting to analyze the hole dynamics (Fig. 3c and Fig. S27), and the results are summarized in Table S8. A fast *τ*_1_ component (where *τ* is carrier lifetime) typically relates to an HT process at the D/A interface, whereas a slower *τ*_2_ component is attributed to diffusion-mediated exciton dissociation. For devices with increased thickness, the fraction of interfacial exciton dissociation increases, leading to enhanced HT efficiency. For the films processed by MID, the fraction of diffusion-mediated exciton dissociation increased significantly, facilitating HT. We attributed this to the films with PP resulting in a significant increase in the ordered acceptor phase, as discussed above.

The decay dynamics of LE in various blend films are depicted in Fig. 3d and Fig. S28. Obviously, the films with PP exhibit faster LE decay, which should be attributed to efficient HT and charge separation [22]. Exciton *L_D_* calculations were used to explore the influence of MID on exciton diffusion behaviors, which is crucial for thick-film OSCs. Figure 3e and Fig. S29 present the fluence-dependent decay traces of photon-induced excitons in films [8]. Pump-fluence-dependent TA spectroscopy considers two main quenching channels for the excitons. One involves radiative and non-radiative recombination with an intrinsic exciton lifetime constant (*τ* *=* 1*/k*) at any excitation fluence, whereas the other involves the bimolecular exciton–exciton annihilation (EEA) with a bimolecular decay rate coefficient (*γ*) only at higher pump fluence [35]. The fitting details are presented in Table S9. At an excitation fluence of 0.61 μJ cm^–2^, we fitted the decay curves of the L8-BO solution by setting *γ* = 0, resulting in trap-induced recombination rates (*k*). Additionally, under the pump fluence of 16.12 μJ cm^–2^, upon fitting the decay curves at 895 nm, we obtained the exciton diffusion coefficients (D) of 0.90 × 10^–2^ and 1.29 × 10^–2^ cm^2^ s^–1^ for 500 nm L8-BO and L8-BO with PP (medium) films, respectively. The exciton *L_D_* (${L}_{\mathrm{D}}$= $\sqrt {D\tau } $), increased from 24.32 nm for the L8-BO film to 29.35 nm for the L8-BO with PP (medium) film. This suggests that MID enhances exciton diffusion, which is beneficial for optimizing thick-film devices that require longer exciton *L_D_* to reach the D/A interface.

To further investigate the impact of PP incorporation on the exciton dynamics channels, we performed global fitting of the TA kinetics (Fig. 3f–i and Figs S30–S32). There are two channels for the transformation of LE states into other exciton states: one involves the conversion to i-DE states, followed by exciton dissociation into free charges via the i-DE states; the other involves the conversion of LEs into charge transfer (CT) states, which subsequently dissociate into free charges [36]. On the basis of this model, we conducted global fitting and found that the proportion of i-DE states increases with the blending of PP. Previous studies have shown that blending PM6 with small-molecule acceptors weakens the interactions between acceptors, suppressing the interconversion of intramolecular excited states and charge transport within the pure acceptor phase [18]. The introduction of PP effectively optimizes the orderly arrangement of L8-BO, enhancing its crystallinity and expanding fiber size, thereby facilitating the transformation of LE states into i-DE states. Moreover, the increase in the proportion of charge separation (CS) states is accompanied by an accelerated dissociation rate, implying an increase in free charge carriers, which corresponds to enhanced charge mobility and exciton *L_D_*. Combining TA and GIWAXS measurements, CCL and exciton *L_D_* increased simultaneously, demonstrating improved L8-BO packing and exciton delocalization. Thus, the larger i-DE fraction observed in complete blends reflects a synergistic effect: PP simultaneously modifies phase separation and strengthens acceptor packing, both of which promote exciton delocalization and prolong exciton *L_D_*.

**Figure 4.**
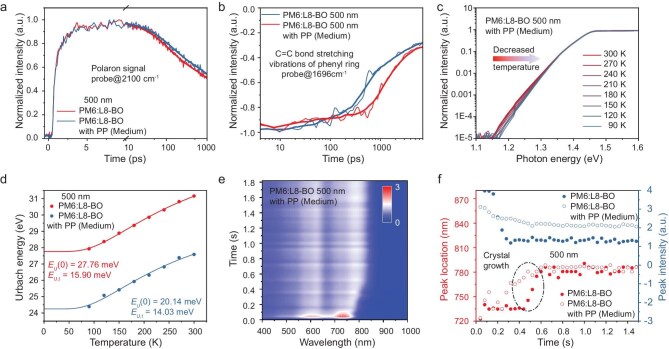
The corresponding TRIR spectra at different delay times. TRIR profiles probed at (a) 2100 cm^−1^ and (b) 1696 cm^−1^. (c) Temperature-dependent sensitive EQE spectra of 500 nm PM6:L8-BO with PP (medium). (d) Urbach energy as a function of temperature for different systems with Cody model fittings. (e) Time-dependent contour maps displaying UV–vis absorption spectra changes during spin coating. (f) Comparison of UV–vis absorption peak location (red line) and peak intensity (blue line) of acceptors with different systems.

Armed with the above insights into exciton–vibration coupling, we further advanced our investigations into the vibrational spectral evolution in the 500-nm-thick films by employing transient infrared (TRIR) spectroscopy (Fig. 4a and b). Upon excitation at 800 nm, the film with insulator molecular-weight modulation presents decelerated charge recombination with respect to the control blend; this result is confirmed by the polaron dynamics extracted using the TRIR signals in the wavenumber of 2100 cm^−1^. This region was chosen due to the absence of molecular vibrational signal contributions [13]. We also compare transient kinetics of the weak GSB peak at 1696 cm^−1^; this peak corresponds to the C=C bond stretching vibrations in the phenyl ring of L8-BO, where the signal is almost free of interference by any positive excited-state absorption signal to the lower wavenumber region [13]. The faster dynamics in the blend with PP (medium) are potentially attributed to the rapid dissipation of molecular vibrational energy. The prompt relaxation of molecular vibrations facilitates the minimization of energy transfer between excitons and vibrational modes, thereby effectively reducing non-radiative recombination losses. The findings from the TRIR analysis further support and cross-validate the results from the TA and temperature-dependent TRPL analyses.

Exciton–vibration coupling significantly influences the energy disorder of systems. Therefore, we conducted a temperature-dependent Fourier-transform photocurrent spectroscopy (FTPS) to explore the degradation of device parameters from the perspective of energy disorder [37] (Fig. 4c and d, Figs S33–S34 and Table S10). The overall Urbach energy is described by Equation 4:


(4) \begin{eqnarray*}
{E}_U\!\left( T \right) = {E}_U\!\left( 0 \right) + \frac{{{E}_{U,t}}}{{{e}^{{\theta }_E/T} - 1}},
\end{eqnarray*}

where *E_U_*(0) represents the static disorder, originating from an intrinsic structural disorder, *E_U,t_* represents dynamic disorder, arising from the thermal motion of molecules at a finite temperature (such as exciton–vibration interactions) and *θ_E_* denotes the Einstein characteristic temperature, corresponding to the mean frequency of lattice phonon excitation. As the thickness of the system increases, the dynamic and static disorder within the systems is significantly amplified, with the enhancement of static disorder being particularly pronounced. Upon the application of the MID with various molecular weights, both the dynamic and static disorder can be substantially mitigated. The suppression of dynamic disorder is primarily attributed to the attenuation of exciton–vibration coupling, whereas the reduction in static disorder stems from the enhanced molecular stacking.

To further understand aggregation changes caused by MID with various molecular weights, *in situ* UV-vis absorption spectra measurements were performed to elucidate the aggregation behavior evolution during the solution-to-solid phase transformation [38] (Fig 4e and f; Figs S35 and S36). As shown in Fig. 4e, the film formation involved three stages. In the first stage, the peak position of L8-BO was almost unchanged, corresponding to the absorption of solution. In the second stage, the position of the absorption peak was observed to be rapidly redshifted, corresponding to the aggregation process during the solution-to-film transformation. In the third stage, the post-film formed and the peak position reached a stable state. We found that the acceptor peak positions of PP-treated blends have a longer duration in the second stage than untreated, suggesting a longer aggregation and crystallization time of the acceptors. We utilized film-depth-dependent light absorption spectroscopy (FLAS) to analyze vertical phase separation between donor and acceptor materials in OSC films of various thicknesses (Figs S37–S40), which is known to significantly influence exciton dissociation and charge transport [35]. An acceptor-rich sublayer exists in the active layer with PP closer to the cathode than in the control active layer, which is beneficial for exciton dissociation and carrier extraction.

**Figure 5.**
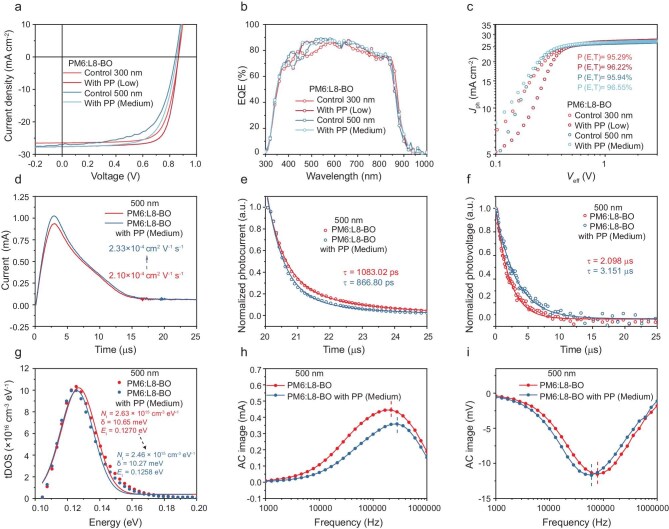
(a) *J–V* curves of different systems. (b) EQE spectra of devices with different systems. (c) The evolution of *J*_ph_ versus *V*_eff_ for the devices. (d) Photo-CELIV curves of the devices. Normalized TPC (e) and TPV (f) data for the related devices. (g) DoS values of blended films and corresponding Gaussian fitting results. IMVS (h) and IMPS (i) curves with different systems.

Representative current density versus voltage (*J–V*) characteristics under simulated AM 1.5G illumination for devices with different thicknesses are shown in Fig. 5a and Fig. S41. Initially, OSCs with an active layer of about 100 nm were fabricated using the PM6:L8-BO BHJ as a control device, yielding a power conversion efficiency (PCE) of 18.17%, aligning with previously published results [39,40]. Detailed parameters are listed in Table S11. Figure 5b and Fig. S42 show the corresponding external quantum efficiency (EQE) spectra. However, upon incorporating PP with different molecular weights into devices with a thickness of 100 nm, the device performance was found to decrease progressively as the molecular weight of the added PP increased. On the contrary, thick-film OSCs incorporating PP with different molecular weights in the photoactive layer exhibited higher PCEs than their control counterparts. Encouragingly, the device with PP (medium) maintained a PCE of 18.06% at an active-layer thickness of 300 nm, exhibiting a short-circuit current density (*J*_SC_) of 27.64 mA cm^−2^, an open circuit voltage (*V*_OC_) of 0.864 V and a fill factor (*FF*) of 75.61%. The maximum PCE of systems is reached when the PP ratio is 1%, and the device parameters deteriorate significantly when it exceeds 1% (Fig. S43 and Table S12). In 100 nm devices, the intrinsic insulating property of PP dominates, introducing significant series resistance and impairing charge transport and extraction. This negative effect outweighs the benefits of its slight improvement in molecular order, leading to a reduction in device efficiency. In contrast, in thick-film devices (>300 nm), the density of donor–acceptor interfaces increases substantially. While the insulating effect of PP remains, its relative impact on charge transport is less detrimental in a thicker, more robust charge transport network. Simultaneously, its ability to enhance and order the molecular packing of the acceptor becomes the dominant factor, leading to significantly improved exciton dissociation, reduced recombination and an improvement in device performance. When the active-layer thickness increases to 500 and 1000 nm, the PCE decreases to 14.14% and 10.83%, respectively. The 500 nm devices exhibited a PCE of 15.92% when blended with PP (medium), whereas the 1000 nm devices achieved a PCE of approximately 12% under blending conditions with PP (high). Such extreme thickness offers decisive practical advantages for roll-to-roll manufacturing: it widens the coating-thickness tolerance and enhances mechanical robustness under mechanical stress. The results demonstrate that precise molecular weight engineering can effectively mitigate performance degradation at increased thicknesses, promoting a better thickness-tolerance property of the device.

For a deeper understanding of PP impact on various acceptor materials, we constructed PM6:Y6 and PM6:BTP-eC9 devices, both with and without PP incorporation (Fig. S44). Remarkably, introducing various molecular weight PPs into various systems led to a significant enhancement in thickness tolerance, consistent with the efficiency improvement trend observed in PM6:L8-BO-based devices. This observation highlights the influential role of insulator molecular weight in optimizing device performance, underscoring its potential as a key component in achieving superior photovoltaic characteristics.

We also studied the exciton dissociation of the devices by investigating the photocurrent density (*J*_ph_) and effective voltage (*V*_eff_) curves, which were used to evaluate the charge-dissociation probability [*P (E, T)*] [41] (Fig. 5c and Fig. S45). As the active-layer thickness increases, the exciton dissociation rate of the devices increases, due to the increase in D/A interfaces. The devices with different thickness exhibited higher exciton dissociation rates after blending with corresponding molecular weight PPs, resulting in increased *J_SC_*. The photoinduced charger-carrier extraction by linearly increasing voltage (photo-CELIV) was utilized to calculate charge carrier mobilities (Fig. 5d and Fig. S46) [42]. The results suggest that blending with PP (medium) effectively improved the directional transport of electrons and holes toward the cathode and anode, resulting in increased carrier mobility from 2.10 × 10^−4^ to 2.33 × 10^−4^ cm^2^ V^−1^ S^−1^ in 500 nm devices. This trend persisted in devices with 300 nm active-layer thickness.

To gain a deeper insight, we further analyzed the charge extraction properties of different thickness devices at the *J_SC_* condition (where the internal field equals the built-in potential), measured by the transient photocurrent (TPC) technique [43]. As presented in Fig. 5e and Fig. S47, the extraction time of the 300 and 500 nm devices were calculated to be *τ* = 412.09 and *τ* = 1083.02 ps. The carrier extraction time was significantly reduced in the thick film devices with MID, indicating an enhanced carrier extraction capability. Additionally, the charge recombination behavior of different thickness devices is performed by transient photovoltage (TPV) measurements [44] (Fig. 5f and Fig. S48). *τ* is extracted from the fit of the TPV signal, with values of 3.098 and 3.151 µs in 500 nm control and PP (medium)-treated devices, respectively. This observation indicates that the introduction of PP with corresponding molecular weight into active layers of different thicknesses can effectively suppress carrier recombination.

Subsequently, we employed frequency-dependent capacitance spectra measurements to investigate the density of states (DoS) of the trap distribution for OSC devices with different thicknesses [45]. The trap DoS at energy *E_ω_* is related to the derivative of the capacitance with respect to the frequency, as in Equation 5:


(5) \begin{eqnarray*}
{N}_{\mathrm{t}}\!\left( {{E}_{\mathrm{\omega }}} \right) = - \frac{{{V}_{bi}}}{{qW}}\frac{{{\mathrm{d}}C}}{{{\mathrm{d\omega }}}}\frac{{\mathrm{\omega }}}{{kT}},
\end{eqnarray*}

where *V_bi_* is the built-in potential and *W* is the width of the space charge region. A Mott–Schottky plot is used to extract the *V_bi_* and the apparent blending profile *N_ap_* [45]. The results are shown in Fig. 5g; Figs S49 and S50, in which the 500 nm device possesses a widely distributed DoS with an *N*_t_ of 2.63 × 10^15^ cm^−3^ eV^−1^ and an energetic disorder of 10.65 meV, while the PP (medium)-treated device exhibits a reduced *N*_t_ of 2.46 × 10^15^ cm^−3^ eV^−1^ and a limited disorder of 10.27 meV.

To survey the relationship between carrier transport and recombination of thick-film devices, we applied intensity-modulated photocurrent/photovoltage spectroscopy (IMPS/IMVS) measurements [46]. The IMPS and IMVS curves are shown in Fig. 5h and i, and Fig. S51. The carrier transport and recombination lifetime can be calculated by *τ* *=* 1/2π × *f_min_*. The detailed *f_min_* values of IMPS and IMVS measurements are listed in Table S13, and transport lifetime *τ_tran_* values were 7.39 and 5.72 × 10^−7^ s, while recombination lifetime *τ_rec_* values were 2.06 × 10^−7^ and 2.65 × 10^−7^ s for 500 nm control and PP (medium)-treated devices, respectively. The enhanced *τ_tran_* and suppressed *τ_rec_* indicate that PP facilitates the faster transport of holes and free charges in thick-film devices, enabling efficient collection prior to recombination. The charge collection efficiency (*η_col_*) was defined by the formula *η_col_* *=* 1 − *τ_tran_*/*τ_rec_*. The device with PP (medium) exhibited an *η_col_* of 78.46%, while it was 64.62% for control devices. Therefore, devices processed using MID exhibit enhanced carrier transfer and collection, along with reduced trap-assisted recombination.

## CONCLUSION

In conclusion, this study demonstrates the significant impact of insulating polymer molecular-weight modulation on the performance of thick-film OSCs due to the polymer swelling effect. By introducing PP with varying molecular weights into PM6:L8-BO systems, we effectively suppressed exciton−vibration coupling, reduced non-radiative recombination and extended exciton *L_D_*. These enhancements enabled substantial improvements in device efficiency while endowing the system with notable thickness tolerance. Consequently, we observed that medium-molecular-weight PP can boost the efficiency of a 500-nm-thick device to 15.92%, which is one of the highest values among 500 nm OSCs, and even when the film thickness reaches 1 μm, the device efficiency can still be maintained at approximately 12%. These findings underscore the critical role of molecular-weight modulation in optimizing thick-film OSC performance and provide valuable criteria for selecting insulator molecular weights to enhance exciton diffusion. This work lays a solid foundation for further advancements in the industrialization of organic photovoltaics, addressing the challenge of poor thickness tolerance in many existing OSC systems, and providing a potential strategy for industrialization.

## Supplementary Material

nwaf387_Supplemental_File
