# Supplementary material for: Suppression of exciton–vibration coupling via modulated insulator dilution for thickness-tolerant organic solar cells
Source: Natl Sci Rev. 2025 Sep 16;12(11):nwaf387. doi: 10.1093/nsr/nwaf387 (PMC12581899; doi:10.1093/nsr/nwaf387)
Supplement: nwaf387_Supplemental_File [file nwaf387_supplemental_file.pdf]

Supplementary data

Suppression of Exciton-Vibration Coupling via Modulated Insulator-Dilution for Thickness-Tolerant Organic Solar Cells

Zhen Fu,[1] Jia-Wei Qiao,[1] Kang-Ning Zhang,[1] Wen-Qing Zhang,[1] Ming-Xu Zhou,[1] Jin-Qun Xu,[1] Peng Lu,[1,2] Hang Yin,[1] Xiao-Yan Du,[1] and Xiao-Tao Hao[1,3,*]

[1] School of Physics, State Key Laboratory of Crystal Materials, Shandong University, Jinan, Shandong, 250100, P. R. China.

[2] School of Physics, National Demonstration Center for Experimental Physics Education, Shandong University, Jinan 250100, China.

[3] School of Chemistry, The University of Melbourne, Parkville, Victoria, 3010, Australia.

E-mail: haoxt@sdu.edu.cn (Xiao-Tao Hao).

Materials

PM6, L8-BO, Y6 and BTP-eC9 were purchased from Solarmer Materials (Beijing) Inc. PEDOT:PSS, Clevios^{TM} P VP AI 4083, was commercially available from Hreraeus. PP with different molecular weights (Mn~4000, 6000 and 8000, corresponding low, medium and high molecular weights) were purchased from Adamas. Chloroform (CF) and 1,8- diiodooctane (DIO) were bought from Sigma-Aldrich. All the materials were used as received without further and purification.

PM6: 9'-(2,12-di-tert-butyl-5,9- dioxa-13b-boranaphtho[3,2,1- de]anthracen-7-yl)-9,9''-diphenyl-9H,9'H,9''H- 3,3':6',3''-tercarbazole

Y6:2,2'-((2Z,2'Z)-((12,13-bis(2-ethylhexyl)-3,9-diundecyl-12,13-dihydro-[1,2,5]thiadiazolo[3,4-e]thieno[2'',3'':4',5']thieno[2',3':4,5]pyrrolo[3,2-g]thieno[2',3':4,5]thieno[3,2-b]indole-2,10-diyl)bis(methanylylidene))bis(5,6-difluoro-3-oxo-2,3-dihydro-1H-indene-2,1-diylidene))dimalononitrile

BTP-eC9:2,2'-[[12,13-Bis(2-butyloctyl)-12,13-dihydro-3,9-dinonylbisthieno[2'',3'':4',5']thieno[2',3':4,5]pyrrolo[3,2-e:2',3'-g][2,1,3]benzothiadiazole-2,10-diyl]bis[methylidyne(5,6-chloro-3-oxo-1H-indene-2,1(3H)-diylidene)]]bis[propanedinitrile]

L8-BO:2,2'-((2Z,2'Z)-((12,13-bis(2-ethylhexyl)-3,9-(2-butyloctyl)-12,13-dihydro-[1,2,5]thiadiazolo[3,4-e]thieno[2'',3'':4',5']thieno[2',3':4,5]pyrrolo[3,2-g]thieno[2',3':4,5]thieno[3,2-b]indole-2,10-diyl)bis(methanylylidene))bis(5,6-difluoro-3-oxo-2,3-dihydro-1H-indene-2,1-diylidene))dimalononitrile

Fabrication of OPV cells

The device structure of ITO/PEDOT:PSS/active layer/PDINN/Ag were used in this work. Patterned ITO substrates (R_{sheet} = 15 Ω per square) were cleaned in sequence in detergent, deionized water, acetone, and isopropanol in an ultrasonic bath for 20 min respectively. Then, the ITO substrates were treated by ultraviolet–ozone for 15 min. The PEDOT:PSS solution (PEDOT:PSS:deionized water is 1:1) was spin cast onto ITO with 4000 rpm for 50 s to as the hole transportation layer, followed by annealing at 150 ℃ for 10 min in the air. For all the devices, the active layers were formed by spin coating a chloroform solution of active layer in a N_2-filled glove box. The total D/A weight ratio was kept at 1:1.2 in BHJ processed devices. For BHJ processed devices, the PM6 concentrations are 8 mg/ml, 12 mg/ml, 16 mg/ml and 20 mg/ml for 100 nm-, 300 nm-, 500 nm- and 1000 nm- thick devices, respectively. 0.25% DIO was used as the solvent additive for these mixed solutions. Subsequently, the films were annealed under 100 ℃ for 10 min. After the annellation, PDINN methanol solution (2 mg mL^{-1}) was spin-coated onto the BHJ at 3000 rpm. Then,Ag cathode (100 nm) was evaporated on the electronic transport layer at a high vacuum of 2×10^{-4} Pa. The effective area of OPV cells working under AM 1.5 G is 6.3 mm^2.

.

Measurement of OPV Device

The current density–voltage (J–V) curve was measured under AM 1.5 illumination of 100 Mw cm^{-2} by Keithley 2400 source meter unit. More than 10 devices were fabricated to ensure the reproducibility of the data. Furthermore, the 7-SCSpec system (Sofn Instrument Co. Ltd.) was used to investigate the EQE of OSCs in air. The LCR meter

IM3536 (HIOKI Co. Ltd.) was used to investigate the Capacitance–Voltage performance of OSCs.

Calculation of Flory−Huggins interaction parameter χ

The definitions of free energy of adhesion and Young's equation are used to deduce Equation (S1)[1]

$$\gamma_l(1+cos\theta) = 2\sqrt{\gamma_s^d\gamma_l^d} + \sqrt{\gamma_s^p\gamma_l^p} \quad \#(S1)$$

where γ_l and γ_s are γ of liquid-vapor and solid-vapor, respectively. γ^p and γ^d are the polar and dispersion components of γ, respectively ($\gamma = \gamma^p + \gamma^d$), and θ is the contact angle.

The Flory-Huggins interaction parameter χ gives a measure of the interaction of the polymer chains with the small molecules as well as the polymer-polymer interaction. We can also use the Flory−Huggins interaction parameter χ to estimate the interaction and miscibility among different components. The χ were calculated between the materials X and Y is employed to describe quantitatively their miscibility as below[2]

$$\chi \propto (\sqrt{\gamma_X} - \sqrt{\gamma_Y})^2\#(S2)$$

where $\gamma_X and\ \gamma_Y$ are the surface tension of the materials (X and Y),respectively.

The Measurements of Photoluminescence (PL) and Time-Resolved Photoluminescence (TRPL)

The PL spectra and TRPL experiments were acquired through a confocal optical microscope (Nanofinder FLEX2, Tokyo Instruments, Inc.) equipped with time-correlated single-photon counting (TCSPC) module (Becker & Hickl, SPC-150). All of the PL spectra were measured using a charge-coupled device (CCD) sensor (DU420A-OE, Andor). The excitation wavelength was fixed at 400 nm. The excitation power of all the PL spectra and TRPL experiments were fixed at 1 μW. The diameter of the laser spot was about 8 μm and its area was calculated as $S=5.02 \times 10^{-7}$ cm^2.

Swelling Effect and Flory-Huggins Theory

Solubility parameter, a key thermodynamic parameter of a substance, makes the prediction of swelling behavior possible and simple. It only dealt with the dispersion forces for the regular solution without polar and associating interactions between polymer and solvent.[3] It can be determined from the enthalpy change as following,

$$\delta = \frac{\Delta H - RT^{\frac{1}{2}}}{V} \tag{S3}$$

where δ is the solubility parameter, ΔH is the heat of vaporization, V is the molar volume, R is the gas constant, and T is the absolute temperature.

It is not sufficient to illustrate the swelling behavior of rubber for the polar system containing polar and hydrogen bonding interactions. Thus, in order to improve the prediction accuracy the three-dimensional solubility parameter (HSP) was introduced by splitting the solubility parameter into three components: dispersion parameter (δ_d), polar parameter (δ_p), and hydrogen bonding parameter (δ_h).[3]

$$\delta_t^2 = \delta_d^2 + \delta_p^2 + \delta_h^2 \tag{S4}$$

This theory is distinguished into two manifestation modes: one-dimensional Flory-Huggins interaction parameter (χ_{1D}) obtained from SP-1D and three dimensional one (χ_{3D}) calculated by SP-3D.[4] The χ_{1D} and χ_{3D} can be obtained by the following Equation:

$$\chi_{1D} = 0.34 + \frac{V}{RT}(\delta_t^R - \delta_t^S)^2 \tag{S5}$$

$$\chi_{3D} = \frac{V}{4RT}(4(\delta_d^R - \delta_d^S)^2 + (\delta_p^R - \delta_p^S)^2 + (\delta_h^R - \delta_h^S)^2) \tag{S6}$$

where the constant term 0.34 is often used, δ_t^R and δ_t^S are the SP-1Ds for rubber and solvent, respectively, δ_d^R, δ_p^R and δ_h^R are the SP-3D for rubber and δ_d^S, δ_p^S and δ_h^S are for solvent, T is the absolute temperature, V is the molar volume of solvent, and R is the gas constant.[4]

Fourier Transform Photocurrent Spectra(FTPS) and Absorption Spectra

The UV-vis absorption spectra were recorded by Hitachi U-4100 spectrophotometer. Sensitive EQE was recorded by PECT-600 Fourier-transform photocurrent spectroscopy.

The measurement of transient absorption (TA) spectrum

The measurement of femtosecond transient absorption spectra were measured with an optical instrument consisting of a Ti:sapphire femtosecond laser (coherent) and an optical parametric amplifier (OPA) system. Then the 800 nm pulse was separated into two parts by a beam splitter. One part was coupled into an optical parametric amplifier (TOPAS, Coherent) to generate the pump pulses at 600 nm. The other part was focused onto a sapphire plate and a YAG plate to generate white light supercontinuum as the probe beams with spectra covering 750-1600 nm. The seed pulses were split into two parts of laser, one for routing to the OPA to provide a 400 and 750 nm pump pulse that were used in this work and the other for generating a broad band of 520–800 nm (visible) and 850–1300 nm (NIR) probe light.

Grazing Incidence Wide-Angle X-ray Scattering (GIWAXS) Measurement.

GIWAXS measurements were performed at the Shanghai Synchrotron Radiation Facility BL16B1 beamline under ambient conditions. X-rays have a wavelength of 1.23984 Å and sample detector distance was calibrated by a silver behenate (AgBH). The angles of grazing incidence for GIWAXS measurements are 0.10 °/0.15 °. Samples were prepared on silicon/poly(3,4-ethylenedioxythiophene) polystyrene sulfonate substrates using identical blend solutions and methods as those used in photovoltaic device fabrication.

The Calculation of Crystalline Coherence Length

GIWAXS characterizes the molecular packing and crystallinity of the active layer. The *d-spacing* associated with the π-π stacking peak indicates the molecular interlayer spacing, which can be calculated from the following equation:

$$d = \frac{2\pi}{q_{z(010)}} \#(S7)$$

In which, $q_{z(010)}$ is the position of the π-π stacking peak in Q space. The results indicate that the π-π stacking spacings of molecules in the (010) direction are comparable in all films. We further calculated the crystalline coherence length (CCL) using the Scherrer equation and quantitatively compared the crystallinity of the blend films:

$$CCL = \frac{2\pi k}{FWHM} \#(S8)$$

In which, k is the shape factor (0.9) and *FWHM* is the full width at half maximum of the π-π stacking peak.[5]

Photo-induced Force Microscopy (PiFM) Image.

For photo-induced force microscopy (PiFM) operation, the first eigenmode of the cantilever is excited by the interaction forces between the sample and the tip, which are induced by the external laser source.[6-7] This interaction is detected in the cantilever mechanics when the laser intensity is modulated at the frequency difference between the first and second eigenmode resonances. In this case, the laser (QCL from Block Engineering) is p-polarized, the pulse width is 20 ns, and the light wavenumber (wavelength) can be tuned between 795 and 1900 cm^{-1}. The probe is a gold-coated tip with a resonance frequency of ~300 kHz. The second eigenmode is used for tip-sample distance stabilization, with a tapping modulation amplitude of a few nanometers and atypical setpoint of between 70 and 80%. When a CW QCL laser (DayLight) is used, the laser is intensity modulated (50/50 duty cycle). The tapping modulation amplitude of the first eigenmode, once the tip is in contact, ranges between 70 and 90 nm, with a typical setpoint of 80%. The second eigenmode of the AFM cantilever is used for PiFM detection.

Atomic Force Microscope (AFM) Image.

AFM measurements were performed on a Bioscope Resolve AFM (Bruker) in a tapping mode under ambient conditions.

The Calculation of Hole Transfer Rate and Efficiency

TA characterizes the hole transfer between the donor and acceptor. To avoid the exciton-exciton annihilation, the pump fluence was kept at low-level of approximately 1 μJ cm^{-2}.The hole transfer rate and efficiency can indicate the change of the trap states density. Donor rising kinetics includes both transfer and acceptor intrinsic decay processes.The hole transfer rate can be can be calculated from the following equation:

$$k_{HT} = k_r - k_0 \#(S9)$$

In which, k_r is the hole transfer rate in blend film and k_0 represents the acceptor intrinsic cavity transfer rate. We further calculated the hole transfer efficiency in the blend films:

$$QE = \frac{k_{HT}}{k_{HT} + k_0} \#(S10)$$

In the above equation, the rate k is the reciprocal of the appearance and disappearance of the fitted GSB signal curve. [8]

The Calculation of trap density(N_t)

The defects density can be calculated form capacitance spectroscopy measurement in dark environment. The frequency axis (f) was transformed into the energy axis (E_ω) as below as the follows

$$E_\omega = kTln\left(\frac{2\nu_0}{\omega}\right) \tag{S11}$$

where ω is the angular frequency calculated by $\omega = 2\pi f$, ν_0 is the attempt-to-escape frequency of 109 Hz, k is the Boltzmann constant, T is the thermodynamic temperature. [9]

Trap density (N_t) at energy E_ω was obtained from the derivative of the measured capacitance relative to the frequency described by

$$N_t(E_\omega) = -\frac{V_{bi}}{qd}\frac{dC}{d\omega}\frac{\omega}{kT} \tag{S12}$$

where d is the thickness of the active layer and V_{bi} is the built-in potential calculated from the Mott-Schottky plot. Then the energy distribution for the density of states (DoS) can be described with Gaussian shape distribution

$$N_t(E) = \frac{N_t}{\sqrt{2\pi}\sigma}\exp\left[-\frac{(E_t - E)^2}{2\sigma^2}\right] \tag{S13}$$

where N_t is the total density, E_t is the center of the DoS, σ is the disorder parameter.

The Film-depth-dependent Light Absorption Spectroscopy (FLAS)

The FLAS spenctra was aquired upon a film-depth dependent light absorption spectrometer (PU100, Puguangweishi Co. Ltd). In-situ oxygen plasma etching at low perssure was used to extract the depth-resolved absorption spectrum for the organic active layer, which was also reported in other previous works.[10-11] According to the law of Beer-Lambert:

$$A = -\log\left(\frac{I_T}{I_0 - I_R}\right) \#(S14)$$

where A is the optical density, I_0 is the incident light density, I_R is the reflection light intensity and I_T is the transmission light intensity. It can be obtained via:

$$I_T = (I_0 - I_R) \times 10^{-A} \qquad (S15)$$

By controlling the etching time, the active layer can be incrementally etched off each sublayer by a soft plasma generated by oxygen glow discharge. Thus, the active layer can be divided into many (sub)layers. The etching process is in-situ monitored by a light absorption spectrometer. Assume that the absorbance of each sublayer is A_1, A_2… and A_n. From this, the total transmitted light intensity can be obtained by:

$$I_T = (I_0 - I_R) \prod_{i=1}^{n} 10^{-A_i} = (I_0 - I_R) \times 10^{-\sum_{i=1}^{n} A_i} \#(S16)$$

Then, we can get:

$$A = A_1 + A_2 + A_3 + \cdots A_n \#(S17)$$

The absorbance of the whole active layer is the sum of absorbance of all sublayers. Consequently, we can get the absorption spectra of all the sub-layers at different depths of active laye.

The Film-depth-dependent Light Absorption Spectroscopy (FLAS)

The film-depth-dependent light absorption spectroscopy was acquired upon a film-depth-dependent light absorption spectrometer (PU100, Puguangweishi Co. Ltd). In-situ soft plasma etching at low pressure (less than 20 Pa) was used to extract the depth-resolved absorption spectrum for the organic active layer.Beer-Lambert's law was utilized to fit the FLAS results, which were subsequently utilized to fit the exciton generation contour upon a modified optical matrix-transfer approach.

In-situ characterization

Xenon lamps were used as light sources to obtain in-situ transmission absorption spectrum from Maya2000 Pro (Ocean Insight) spectrometer for the blade coating process.

Photocurrent density (J_{ph}) versus effective voltage (V_{eff}) characterization

Photocurrent density (J_{ph}) as a function of the effective voltage (V_{eff}) was measured to investigate photocarrier dynamic process. The J_{ph} is defined as $J_L - J_D$, where J_L and J_D were the current densities under AM 1.5G 100 mW cm^{-2} illumination and in the dark, respectively. The V_{eff} is defined as V_0 -V, where V was the applied voltage, and V_0 was the voltage when J_{ph} = 0. The J_{ph} value will come into the saturated state (J_{sat}) under a relatively large voltage of ~2 V, indicating that almost all excitons can be effectively dissociated into free charge carriers and then extracted by two separate electrodes. The exciton dissociation efficiency and charge collection efficiency can be calculated by J_{ph}/J_{sat} values under short-circuit and maximal power output conditions, respectively.[12]

Molecular dynamics simulation

The parameters of bonded interactions were obtained through quantum chemistry calculation. The structures of the molecules are optimized at B3LYP-D3(BJ)/6-31G(d,p) level[13-15] using Gaussian 16 A.03 software, and vibration analyses are carried out to ensure that the structures have no virtual frequency. The RESP charge[16] is calculated using Multiwfn 3.8(dev) software[17], and the GAFF force field[18] parameters are obtained using acpype code[19]. Molecular dynamics simulations are performed using GROMACS 2021.7 software [20]. Long-range electrostatic interactions are treated with the particle mesh Ewald (PME) method [10][21] with 1.2 nm as the Coulomb cutoff, and the van der Waals (vdW) interactions are treated with the force-switching method [11][22], where the forces smoothly decayed to zero between 0.9 and 1.2 nm to reduce the cutoff noise. The LINCS algorithm [23] is used to apply the bond constraint related to hydrogen, and a dispersion correction is used for both energy and pressure. The quasi-equilibrated system was a mixture of 5000 chloroform and 100 conformations of L8-BO which was extracted during the NPT equilibrated simulation. All the simulation systems are energy-minimized by 5000 steps steepest descent with a time step of 1 fs, and then undergo a 50 ns NPT run using leap-frog MD integrator [24] with a time step of 2 fs. During all the simulations, temperature is kept constant at 298.15K by using the velocity rescaling (V-rescale) thermostat [25] (with τ_t = 1.0 ps), and pressure is kept at 1 bar by C-rescale isotropic barostat (with τ_p = 0.5 ps)[26]. The initial size of the simulated box is 10 nm $\times$ 10 nm $\times$ 10 nm and three-dimensional periodic boundary condition (PBC) is used. Results are visualized using VMD 1.9.3 [27] software.

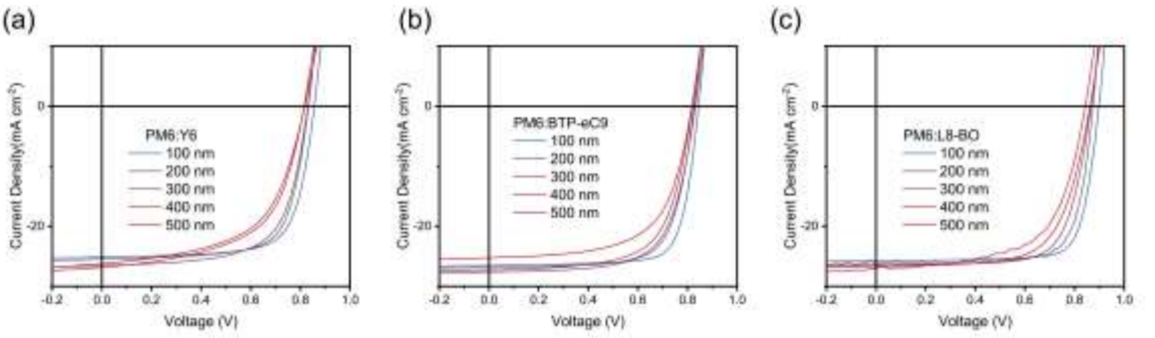

Figure S1. Current density versus bias voltage (*J–V*) curves of the OSCs of (a) PM6:Y6, (b) PM6:BTP-eC9 and (c) PM6:L8-BO with different thickness.

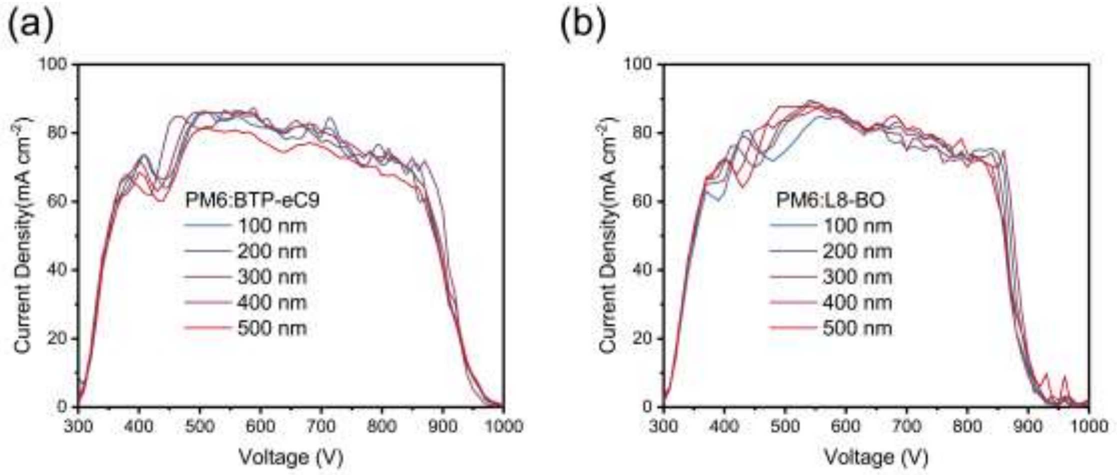

Figure S2. EQE spectra of different thickness with (a) PM6:BTP-eC9 and (b) PM6:L8-BO.

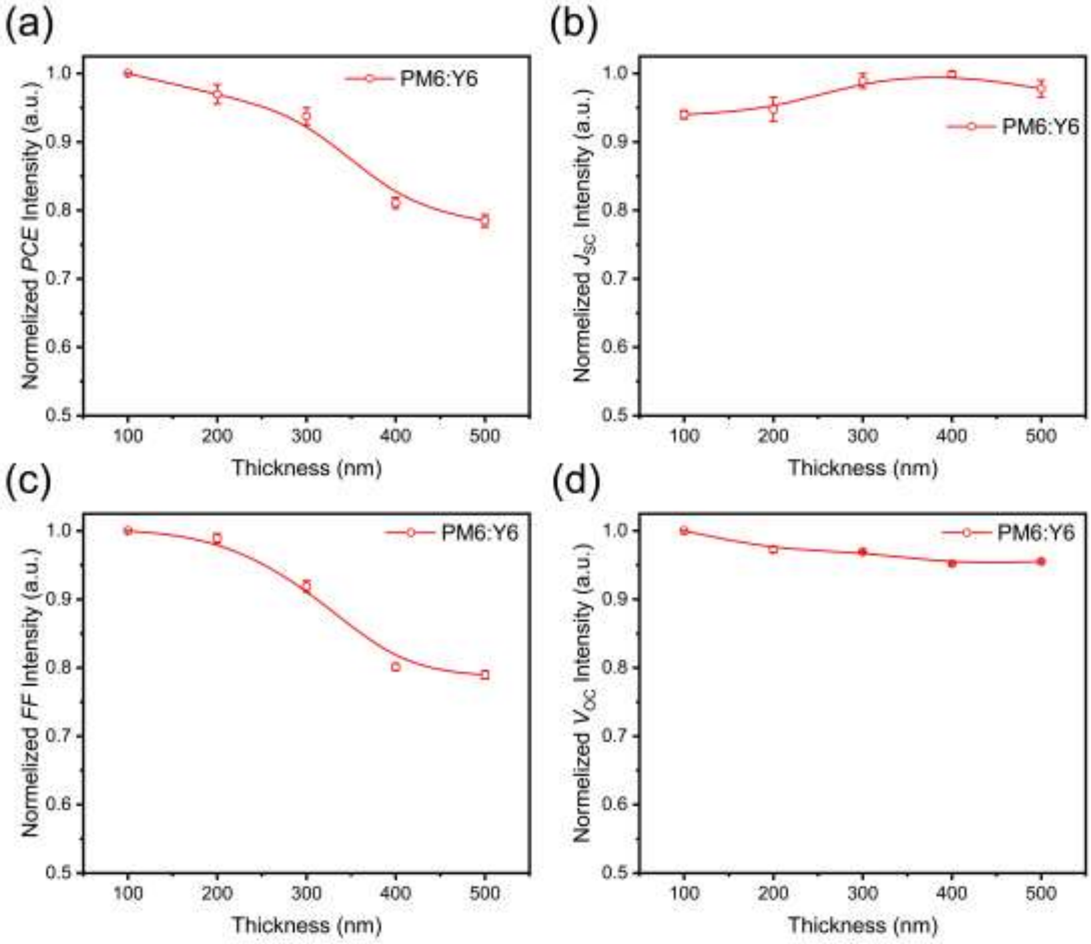

Figure S3. The variation trend of device parameters in PM6:Y6 increases with the active layer thickness.

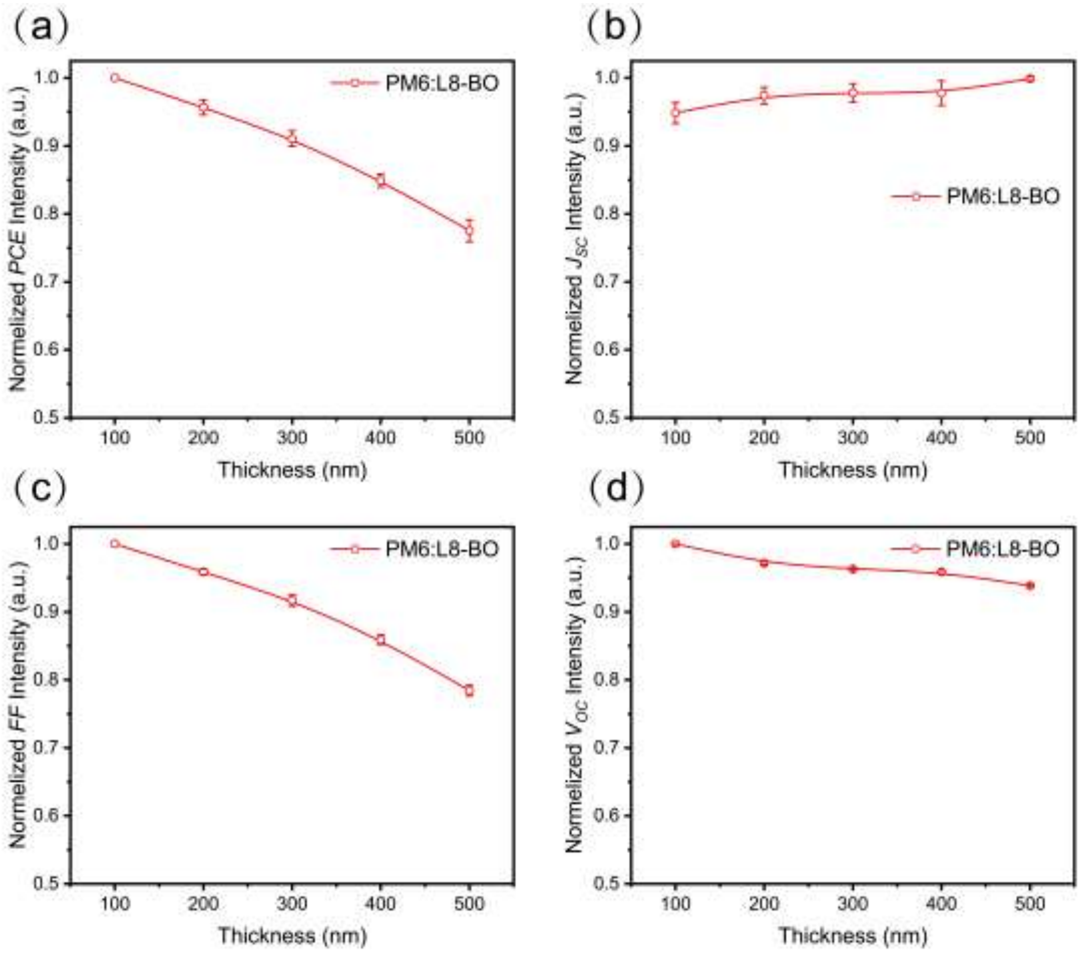

Figure S4. The variation trend of device parameters in PM6:L8-BO increases with the active layer thickness.

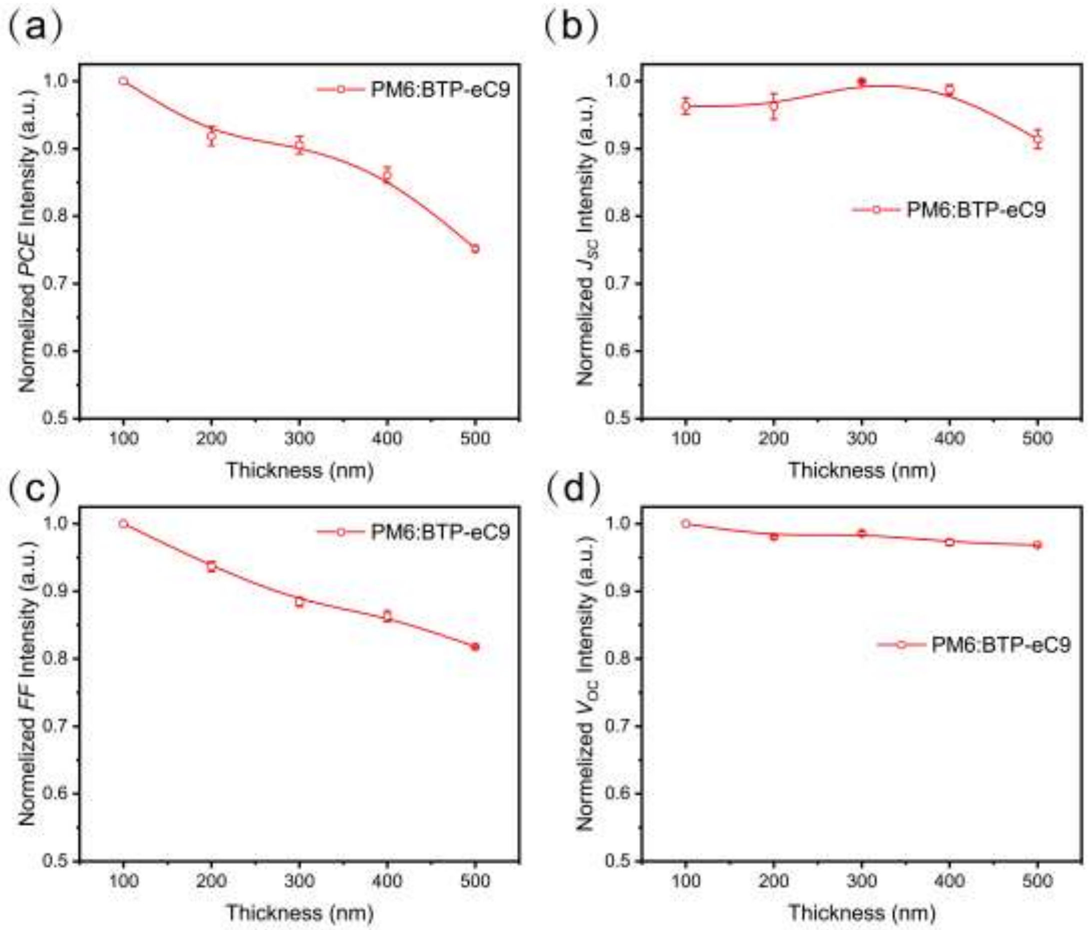

Figure S5. The variation trend of device parameters in PM6:BTP-eC9 increases with the active layer thickness.

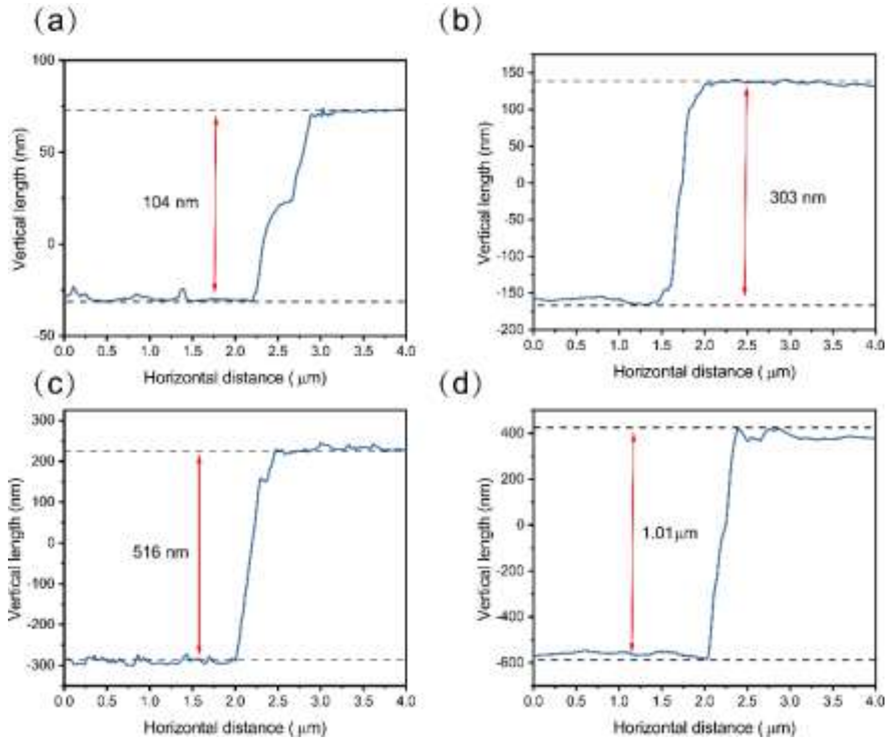

Figure S6. The active layer thickness measured by atomic force microscopy (AFM).

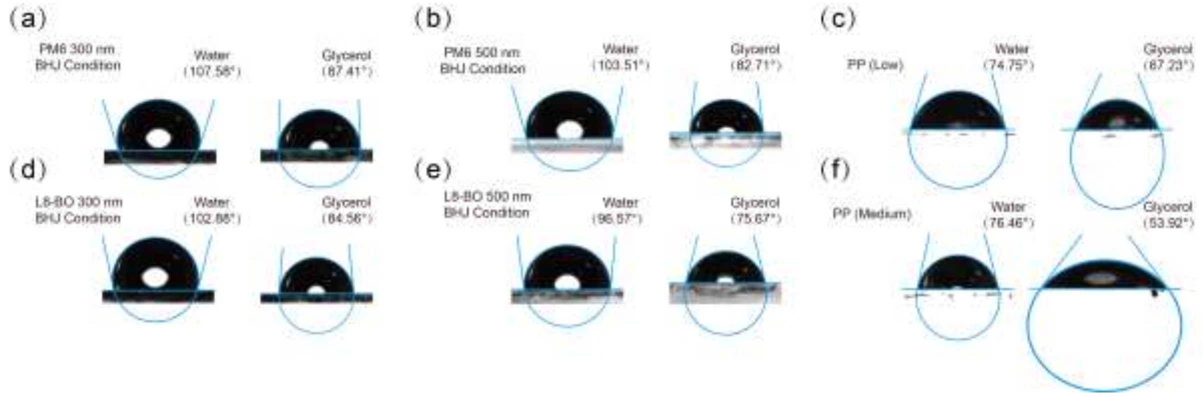

Figure S7. Glycerin and Water contact angle images of different materials.

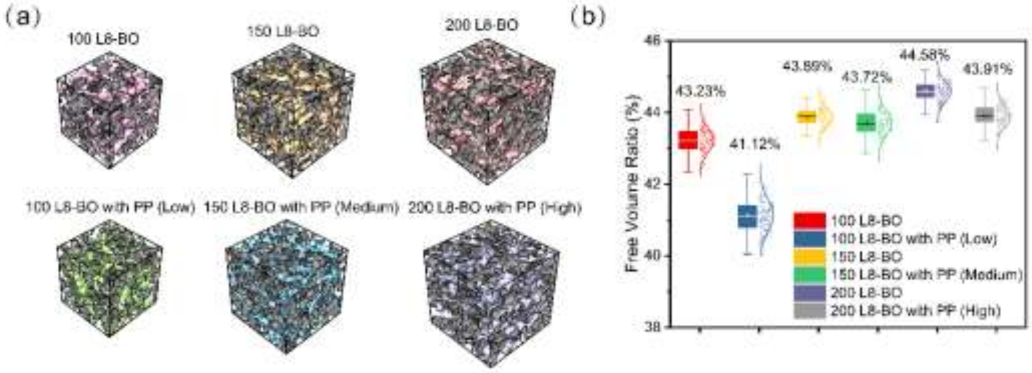

Figure S8. a) Representative snapshots of MD simulated molecular packing morphologies. b) FVR ratio of different systems.

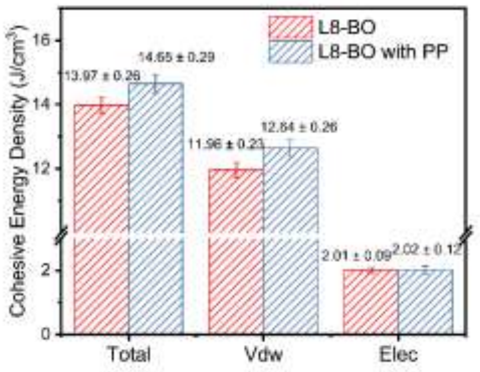

Figure S9. Cohesive energy density (CED) of L8-BO and L8-BO with PP.

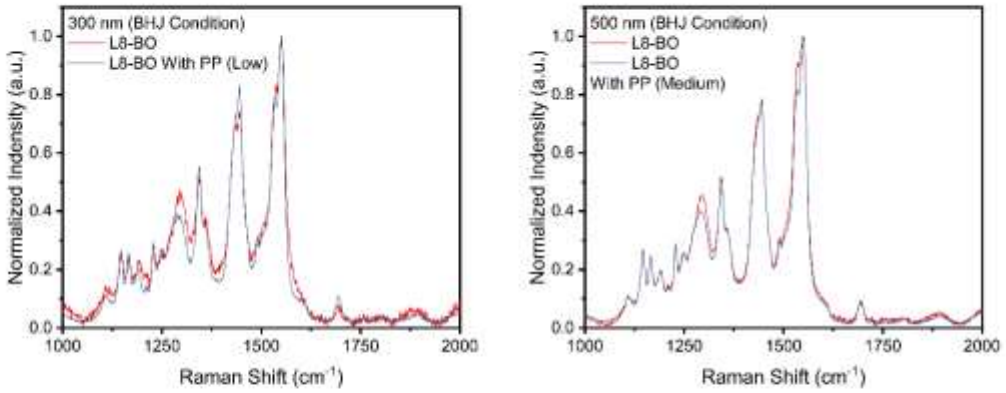

Figure S10. Raman spectra of (a)L8-BO and (b) L8-BO with PP.

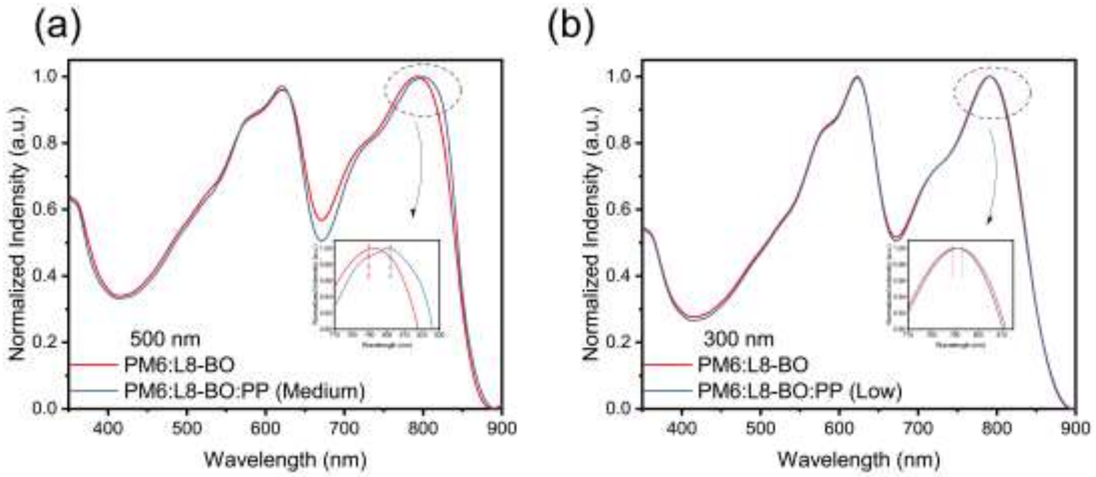

Figure S11. The UV/Vis absorption spectra of 500 nm and 300 nm PM6:L8-BO.

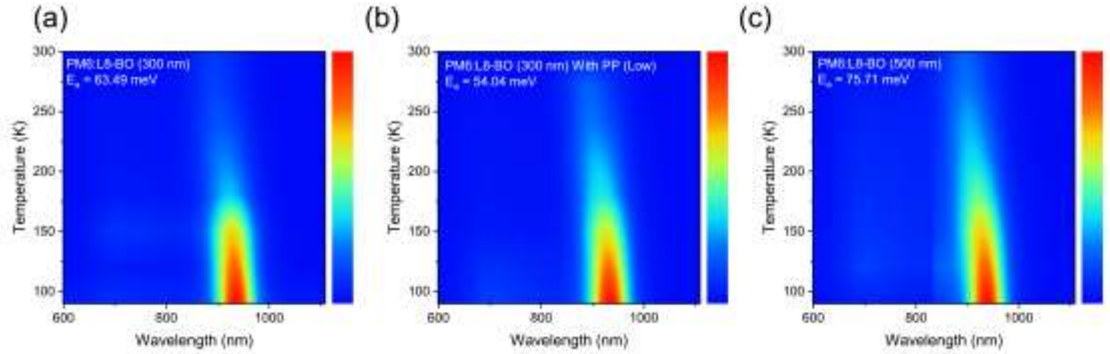

Figure S12. Temperature-dependent photoluminescence spectra of different conditions.

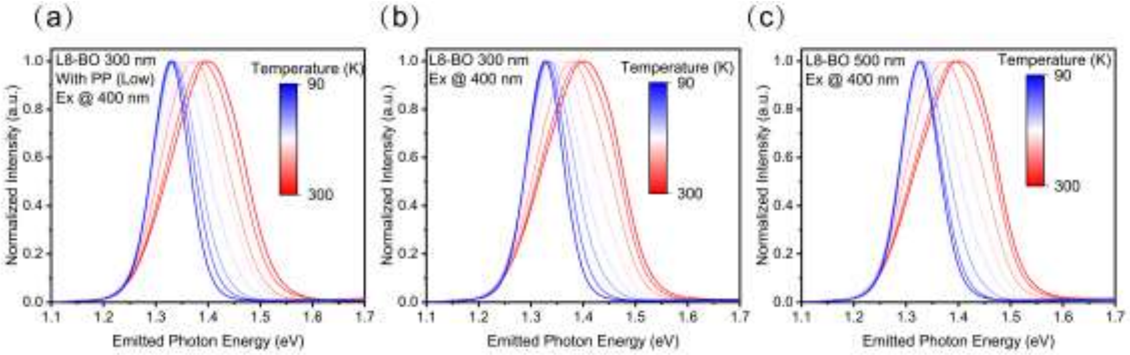

Figure S13. Temperature-dependent photoluminescence spectra of L8-BO with different conditions.

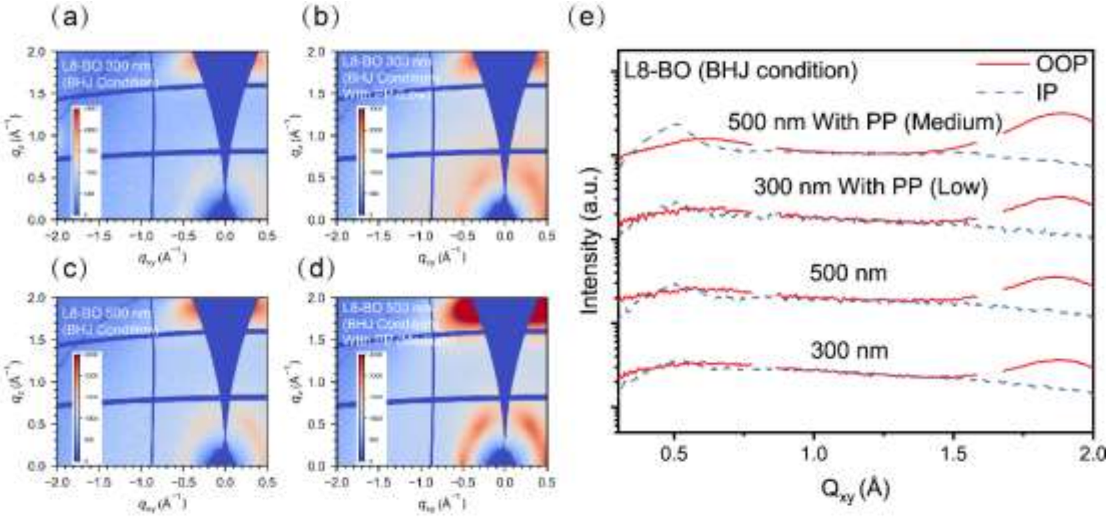

Figure S14. (a)-(d) 2D GIWAXS patterns. (e) Extracted line-cut profiles (IP - dotted line, OOP - solid line) from the corresponding blends.

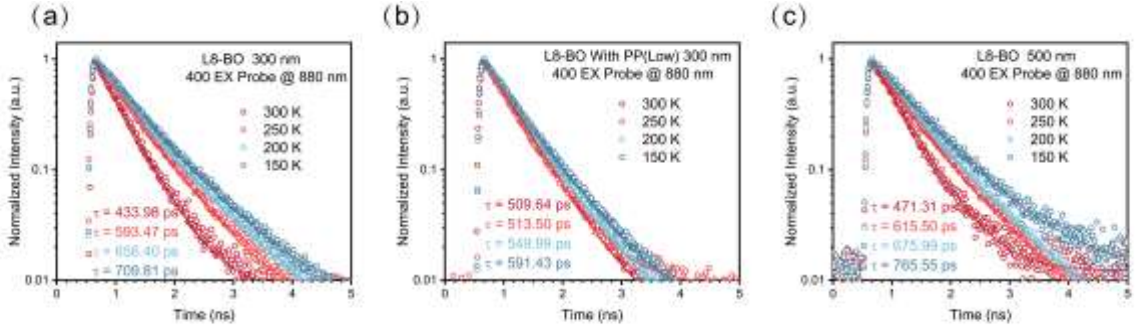

Figure S15. Temperature-dependent TRPL profiles of different blends (pump @ 800 nm).

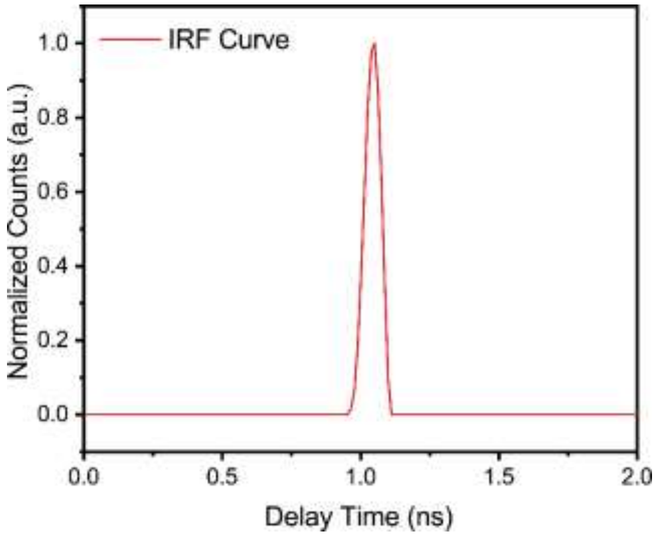

Figure S16. The instrument response function of TRPL measurement.

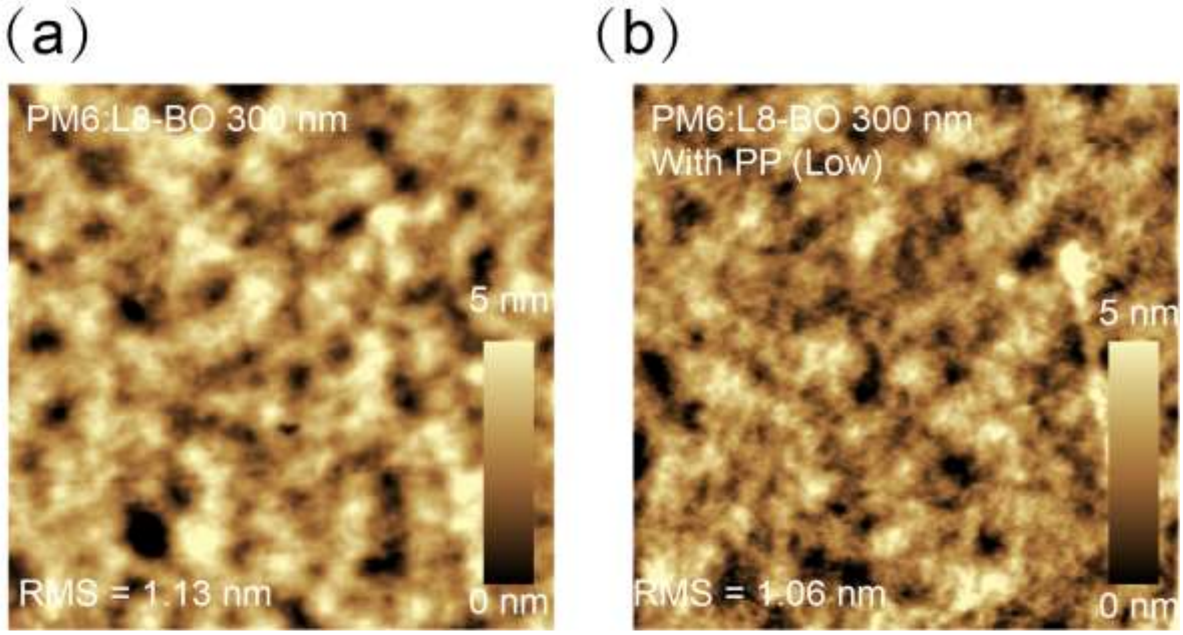

Figure S17. AFM height images of different systems.

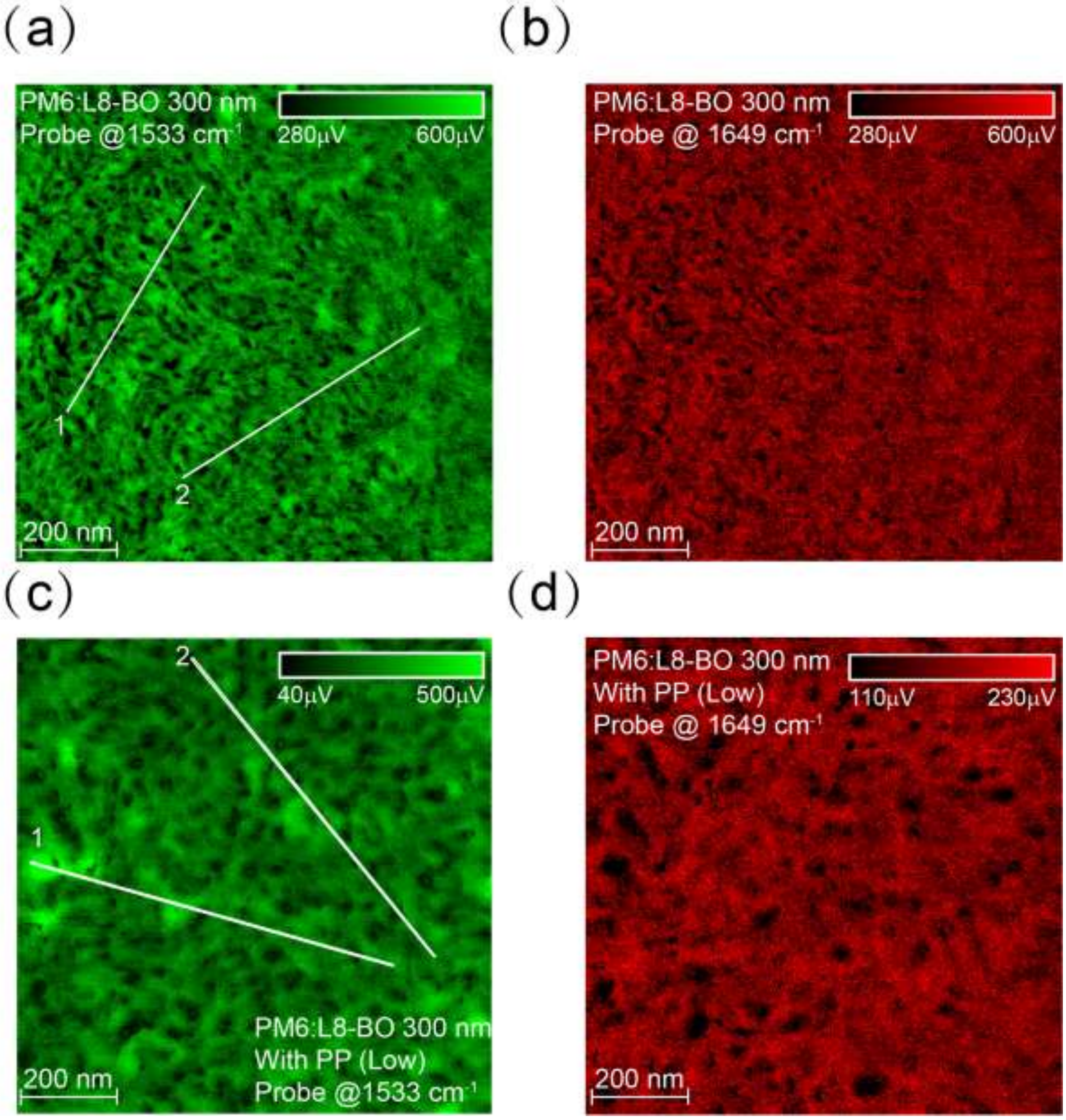

Figure S18. Photo-induced force microscopy images of 500 nm films at a wavenumber of 1533 cm^{-1} (representing L8-BO) and 1,649 cm^{-1} (representing PM6).

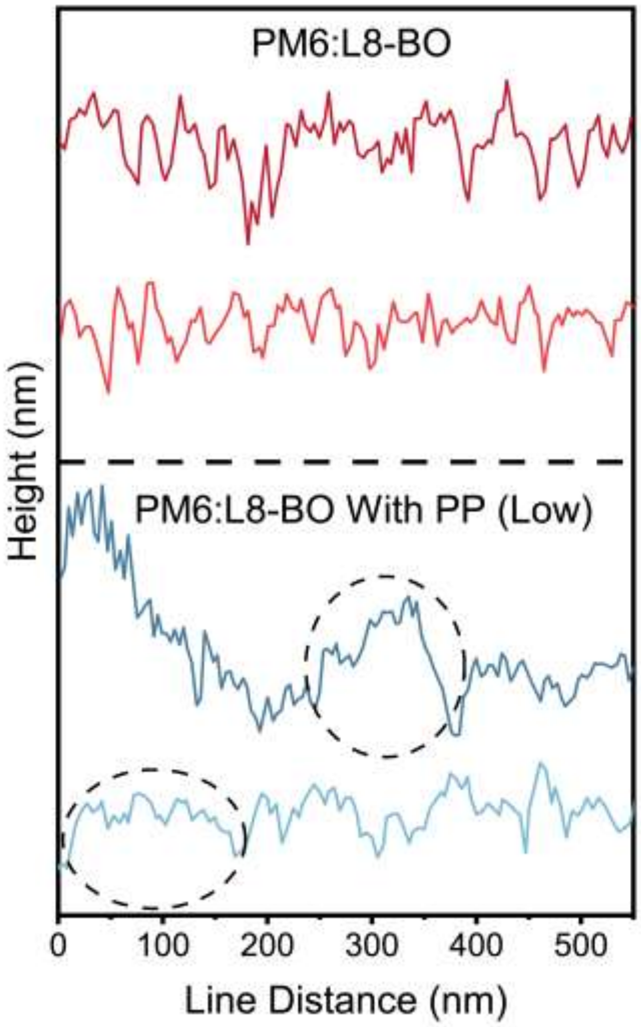

Figure S19. The line profile of nano fiber cross sections obtained by PiFM map.

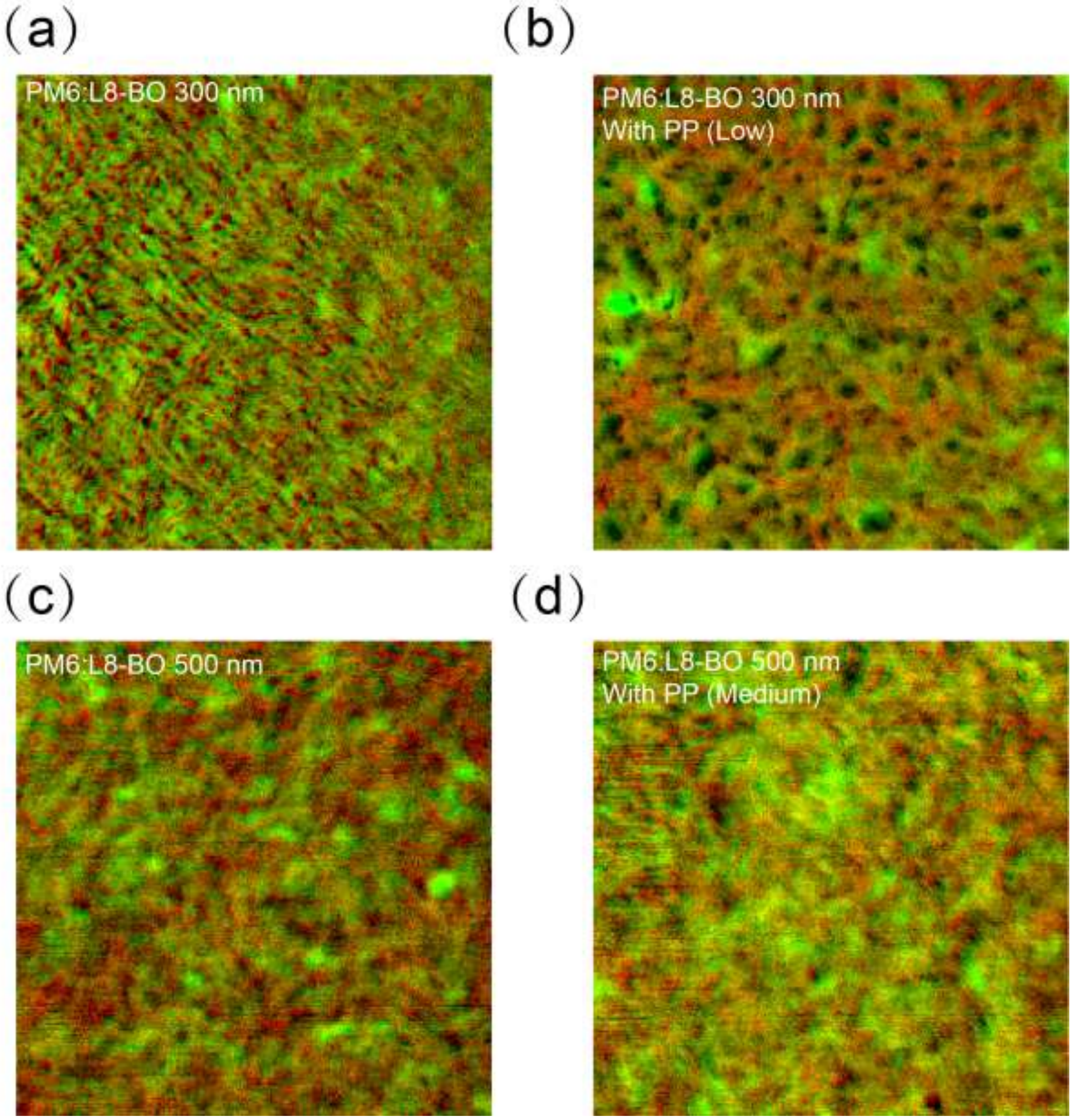

Figure S20. The integration of the PiFM images of the two wavenumbers can segment different areas and perform corresponding percentage calculations.

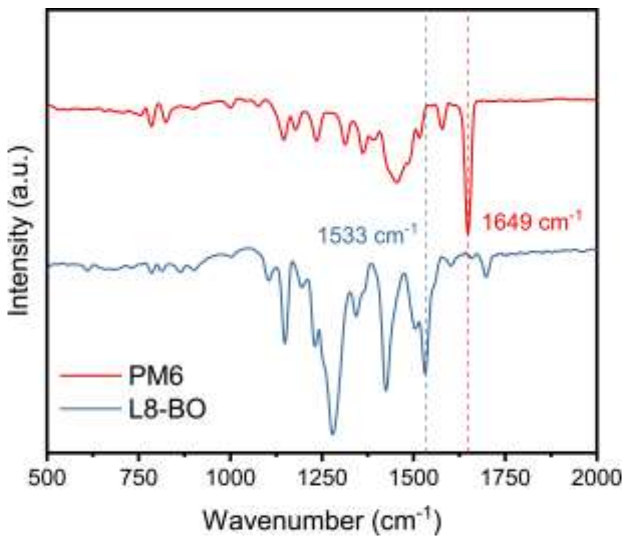

Figure S21. FTIR spectra of PM6 and L8-BO.

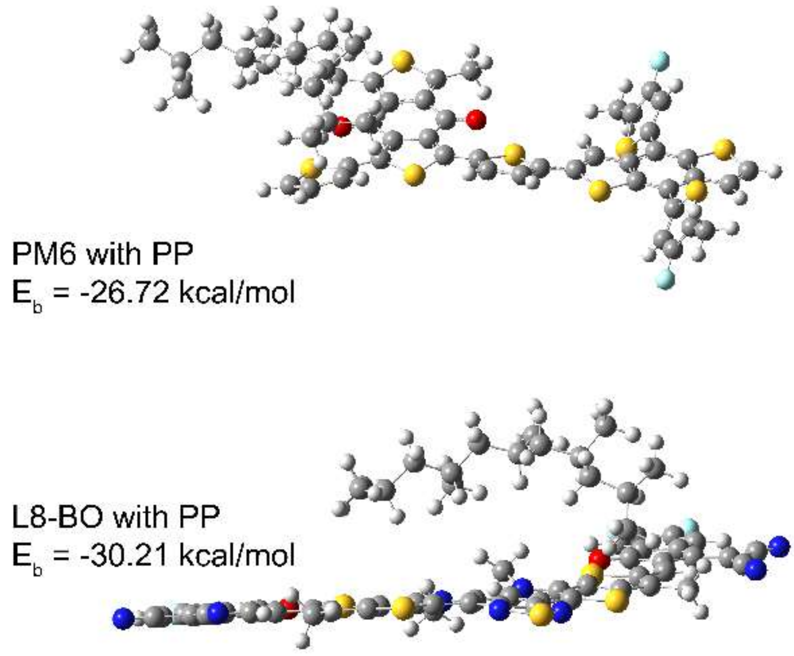

Figure S22. Binding-energy of (a) L8-BO with PP and (b) PM6 with PP.

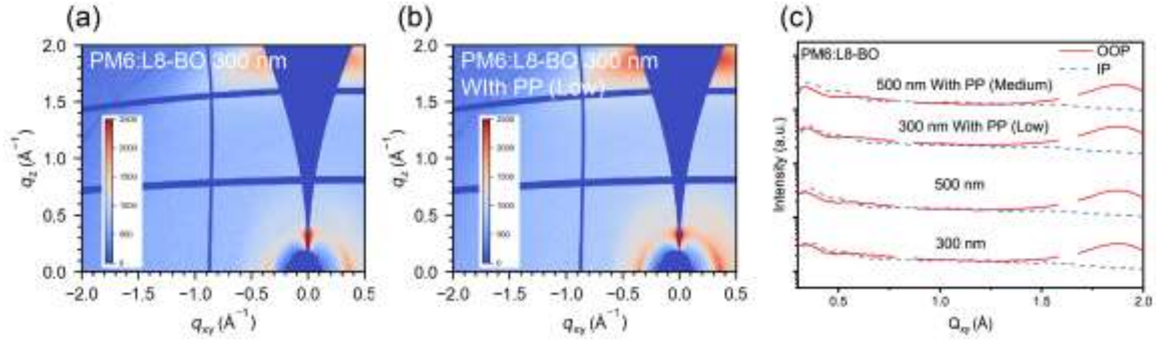

Figure S23. a)-b) 2D GIWAXS patterns. c Extracted line-cut profiles (IP - dotted line, OOP - solid line) from the corresponding blends.

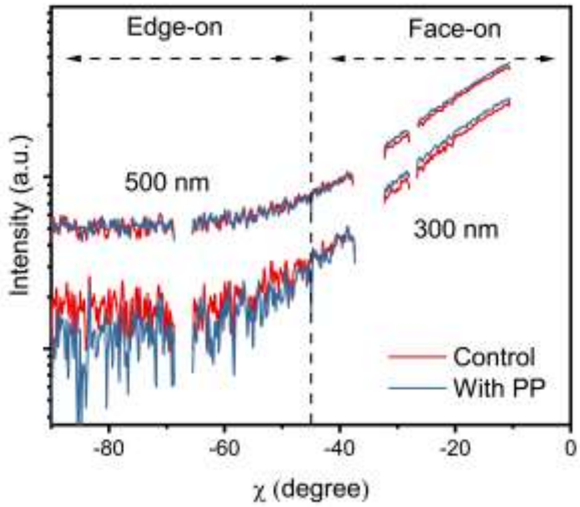

Figure S24. Pole figures extracted from the (010) diffractions for corresponding films. The integrated area of the polar angle χ in the range 0-45 ° (0--45 °) and 45-90 ° (-45--90 °) is defined as the ratio of face-on (light blue area) and edge-on (light pink area) crystallites, respectively.

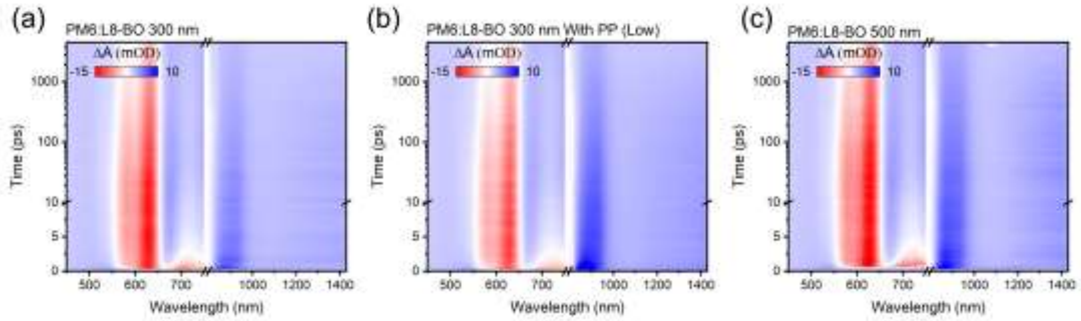

Figure S25. 2D TA spectrum of the film with different systems.

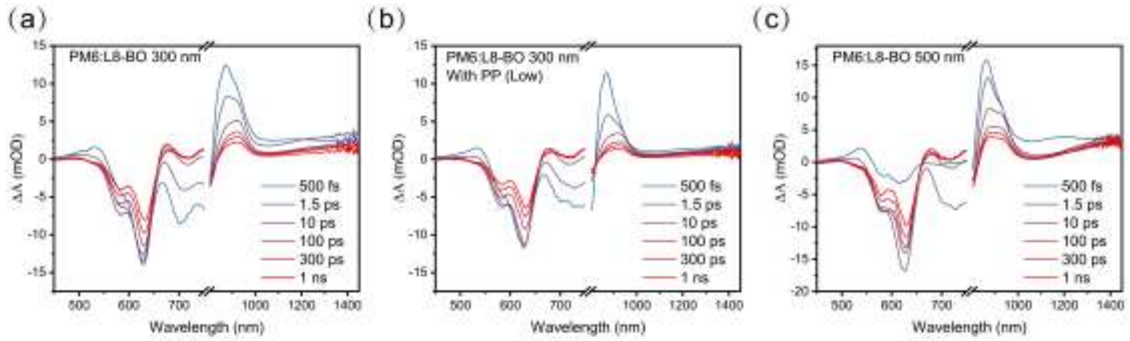

Figure S26. The corresponding TA spectra at different probe delay times with different systems.

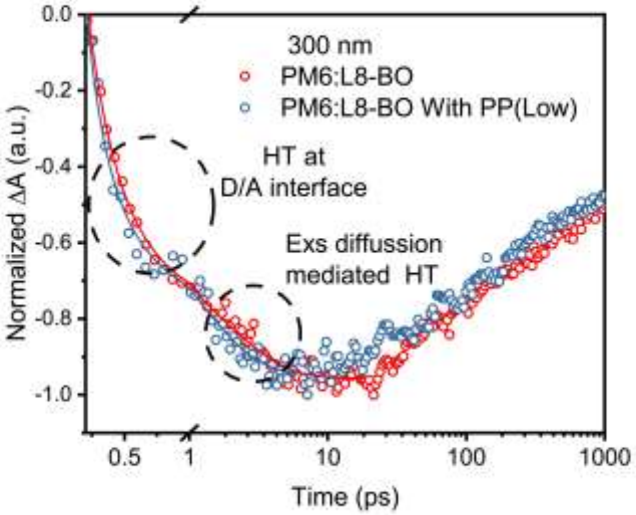

Figure S27. Dynamics of the rising signal of PM6 GSB in different films, with solid curves representing quadratic fitting results.

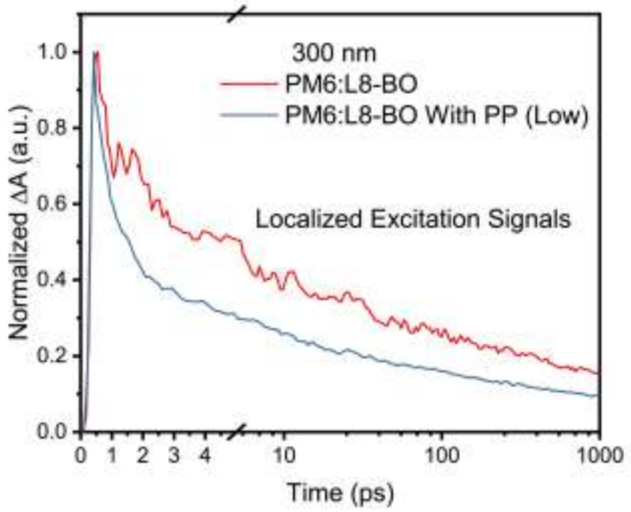

Figure S28. TA dynamics of the LE signals in different films.

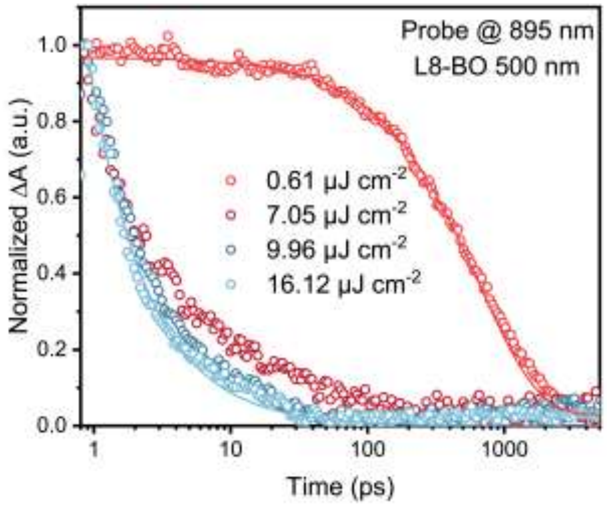

Figure S29. Pump-fluence dependent TA kinetics of neat L8-BO film traced at 895 nm.

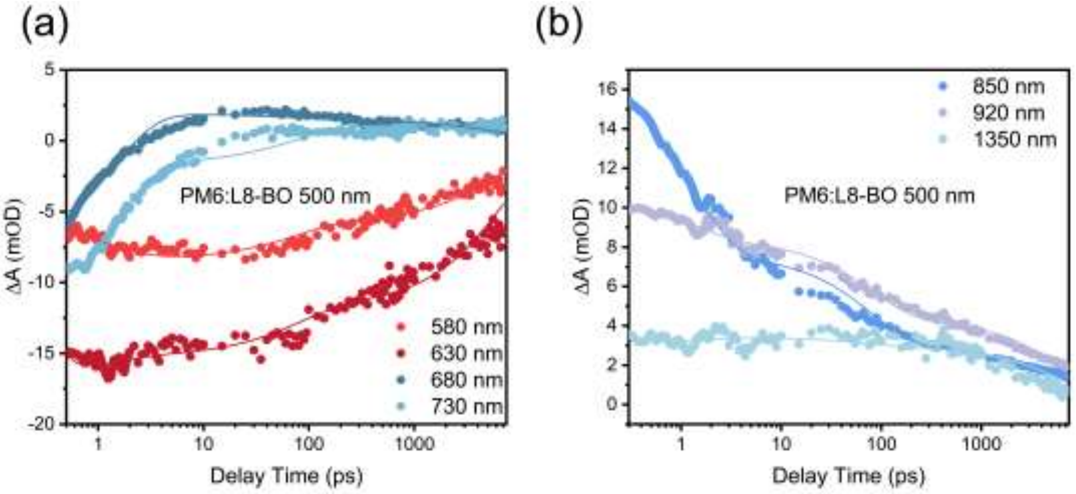

Figure S30. Global fitting analysis of TA data recorded from 500 nm PM6:L8-BO film.

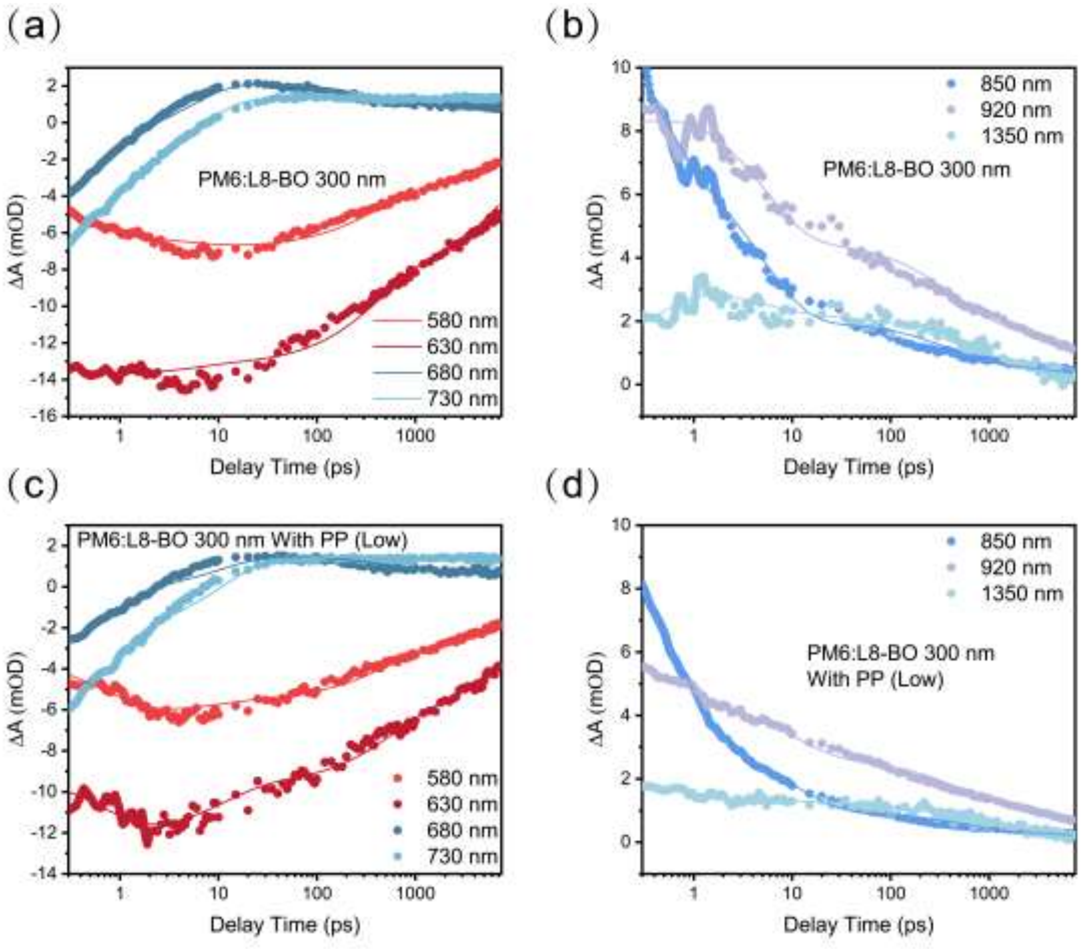

Figure S31. Global fitting analysis of TA data recorded from 300 nm PM6:L8-BO film (a)-(b) with and (c)-(d) without PP (Low).

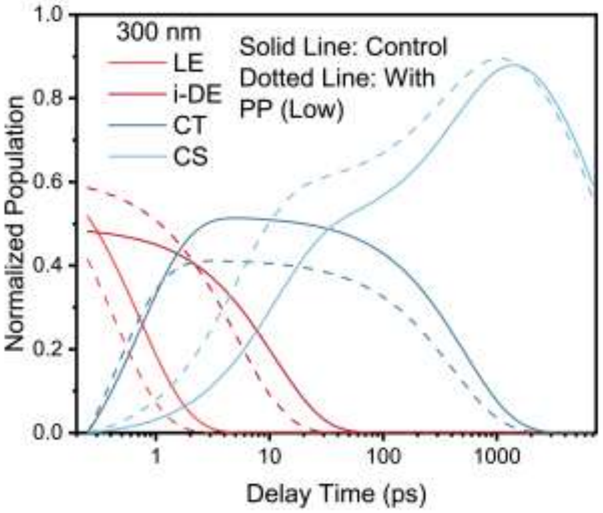

Figure S32. Global fitting analysis of TA data recorded from 300 nm PM6:L8-BO film with different conditions.

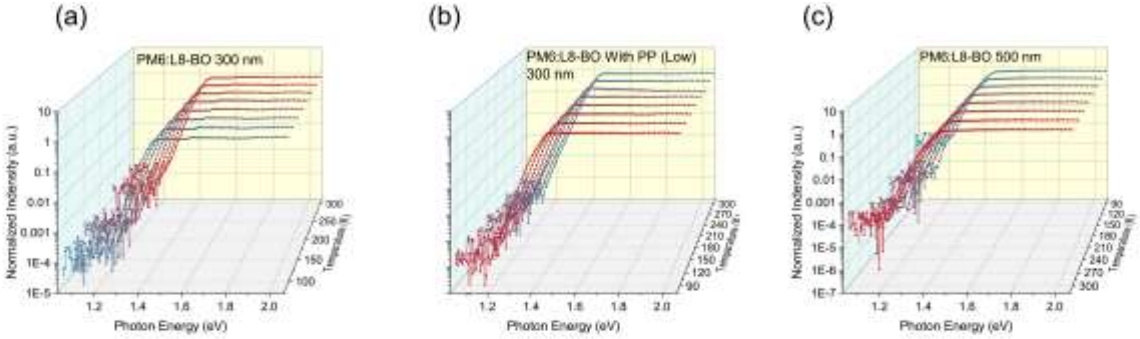

Figure S33. Temperature-dependent Sensitive EQE spectra of devices based on different systems .

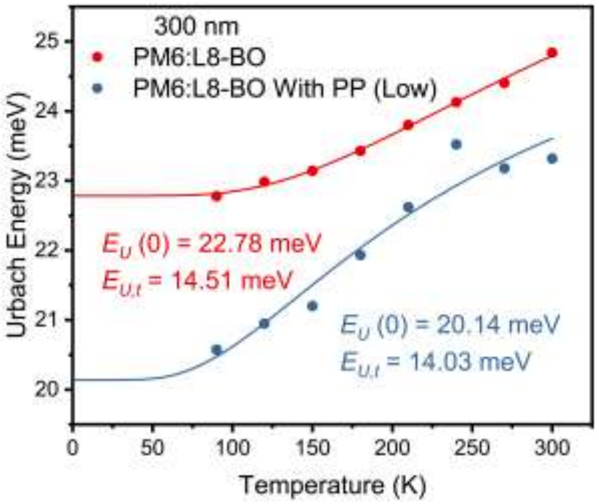

Figure S34. Urbach energy as a function of temperature for different systems with Cody model fittings.

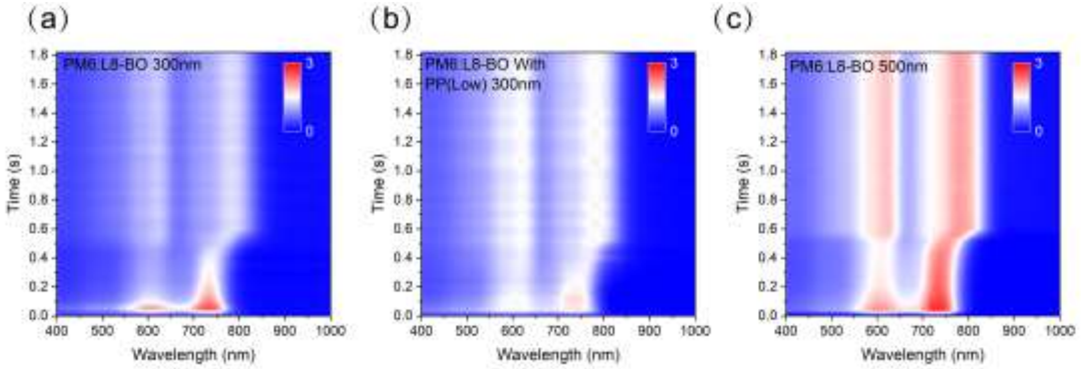

Figure S35. Time-dependent contour maps of UV–vis absorption spectra during spin coating with (a) 300 nm PM6:L8-BO, (b) 300 nm PM6:L8-BO with PP (Low) and (c) 500 nm PM6:L8-BO films.

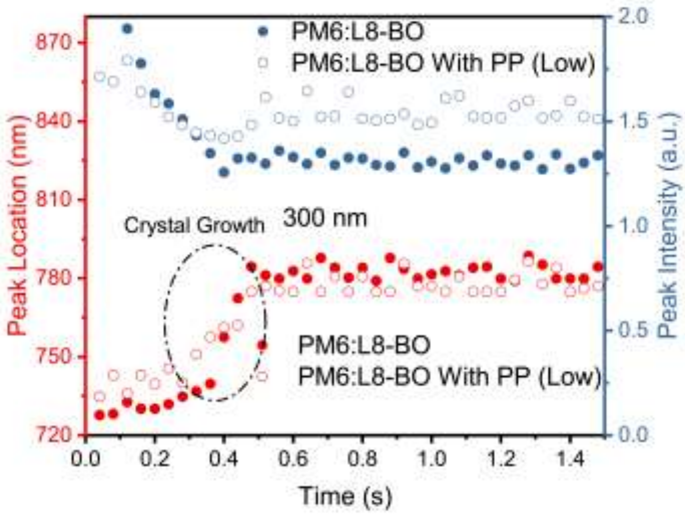

Figure S36. Comparison of UV–vis absorption peak location (red line) and peak intensity (blue line) of acceptors with different systems.

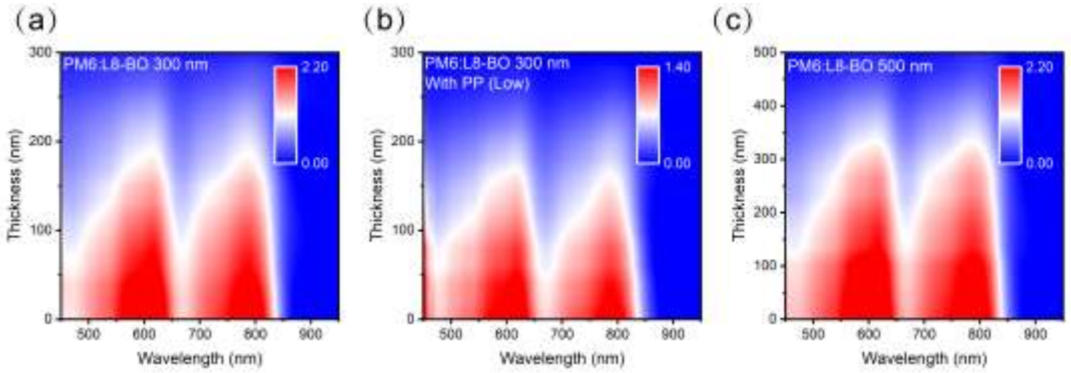

Figure S37. Absorption characteristics of the sub-layers with different systems varied during the FLAS etching process.

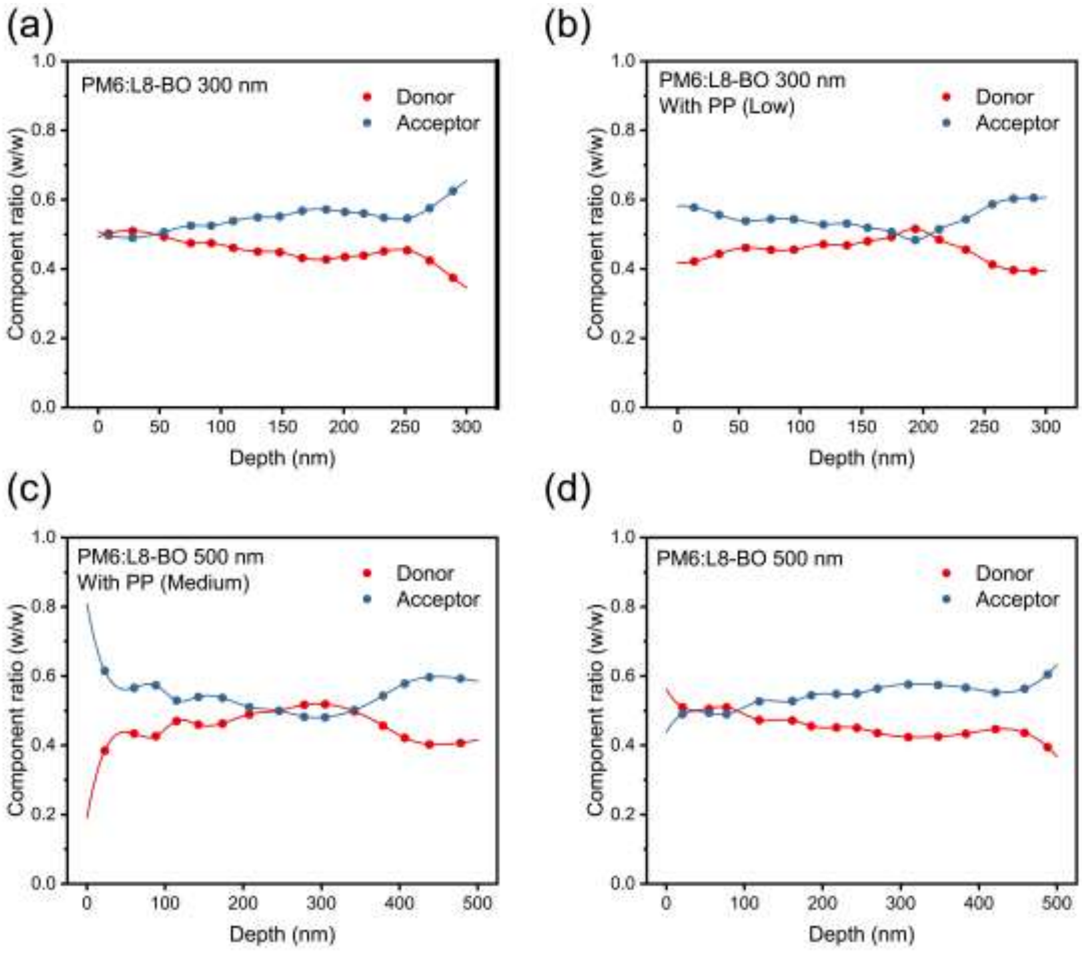

Figure S38. Vertical phase distribution curves of the corresponding films, varied during the FLAS etching process.

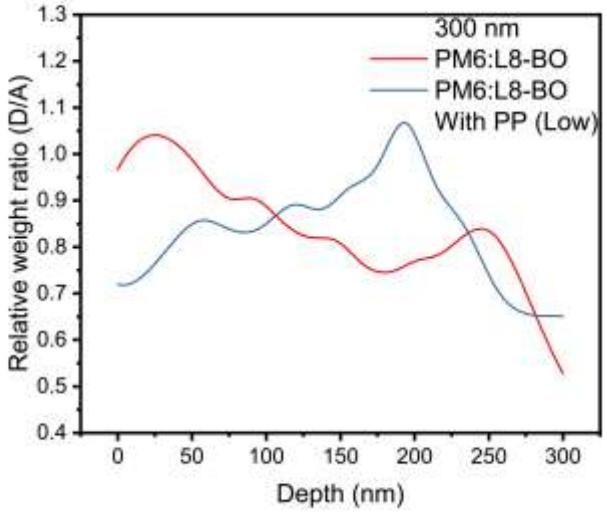

Figure S39. Evolution of vertical phase distribution during degradation.

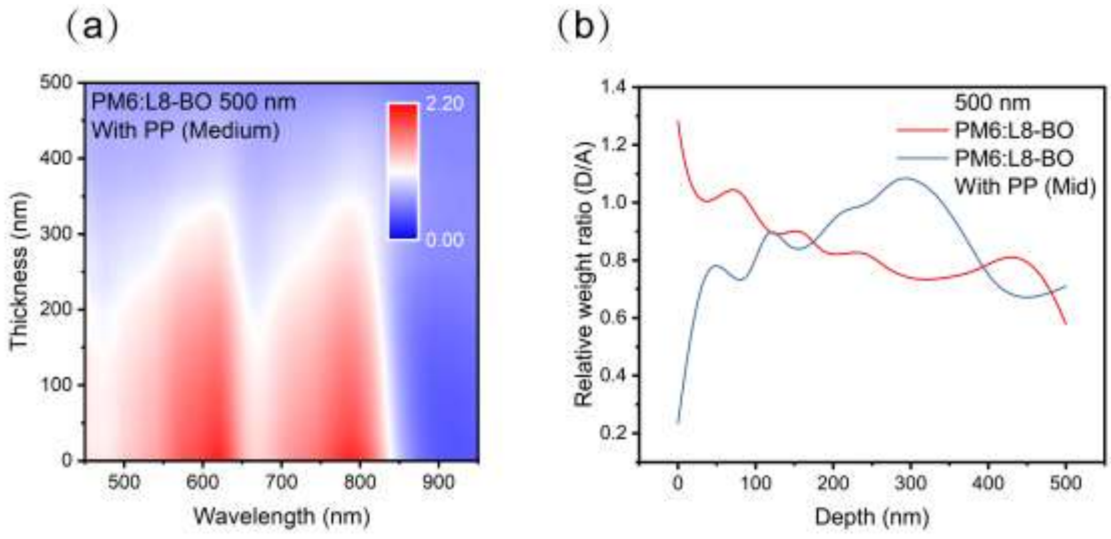

Figure S40. Vertical phase distribution during the FLAS etching process for the corresponding films.

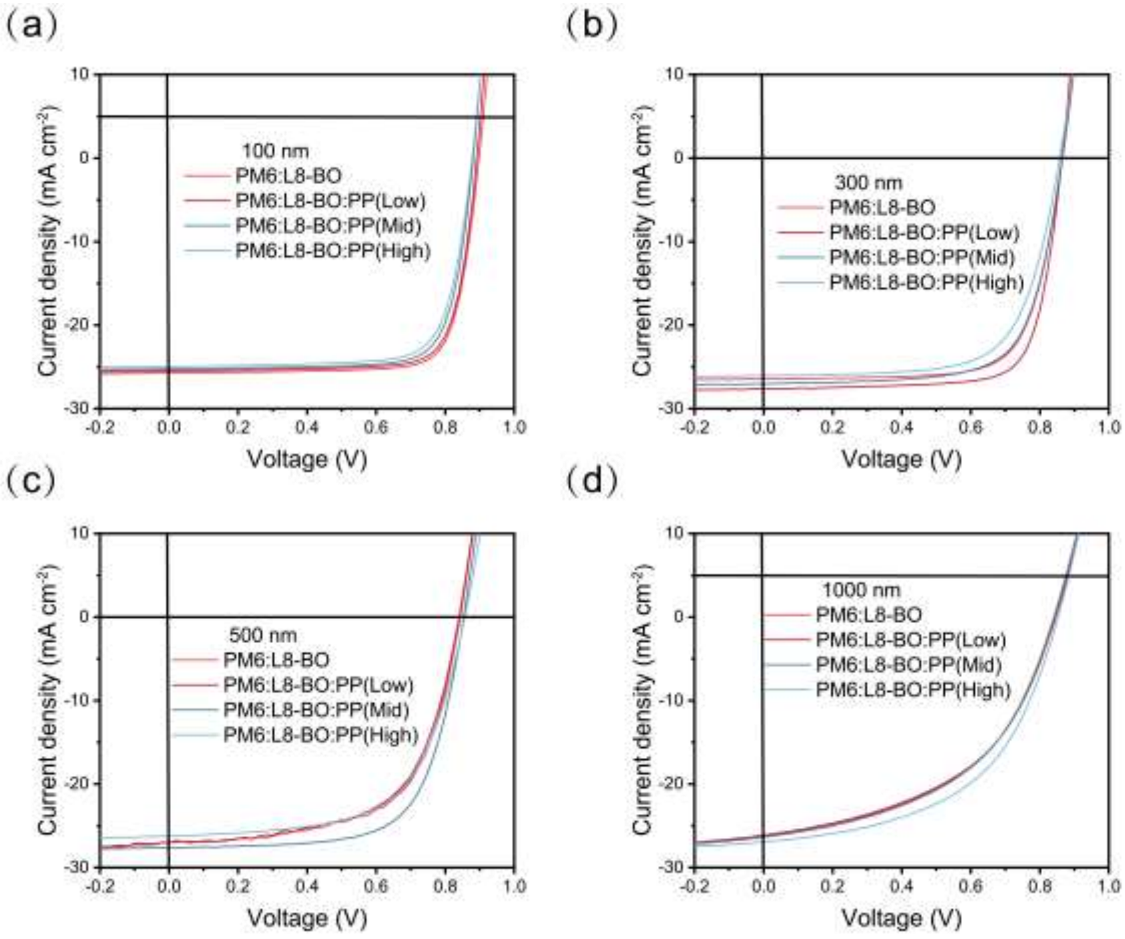

Figure S41. Current density versus bias voltage (*J*–*V*) curves of the OSCs of (a) 100 nm, (b) 300 nm, (c) 500 nm and (d) 1000 nm system with different conditions.

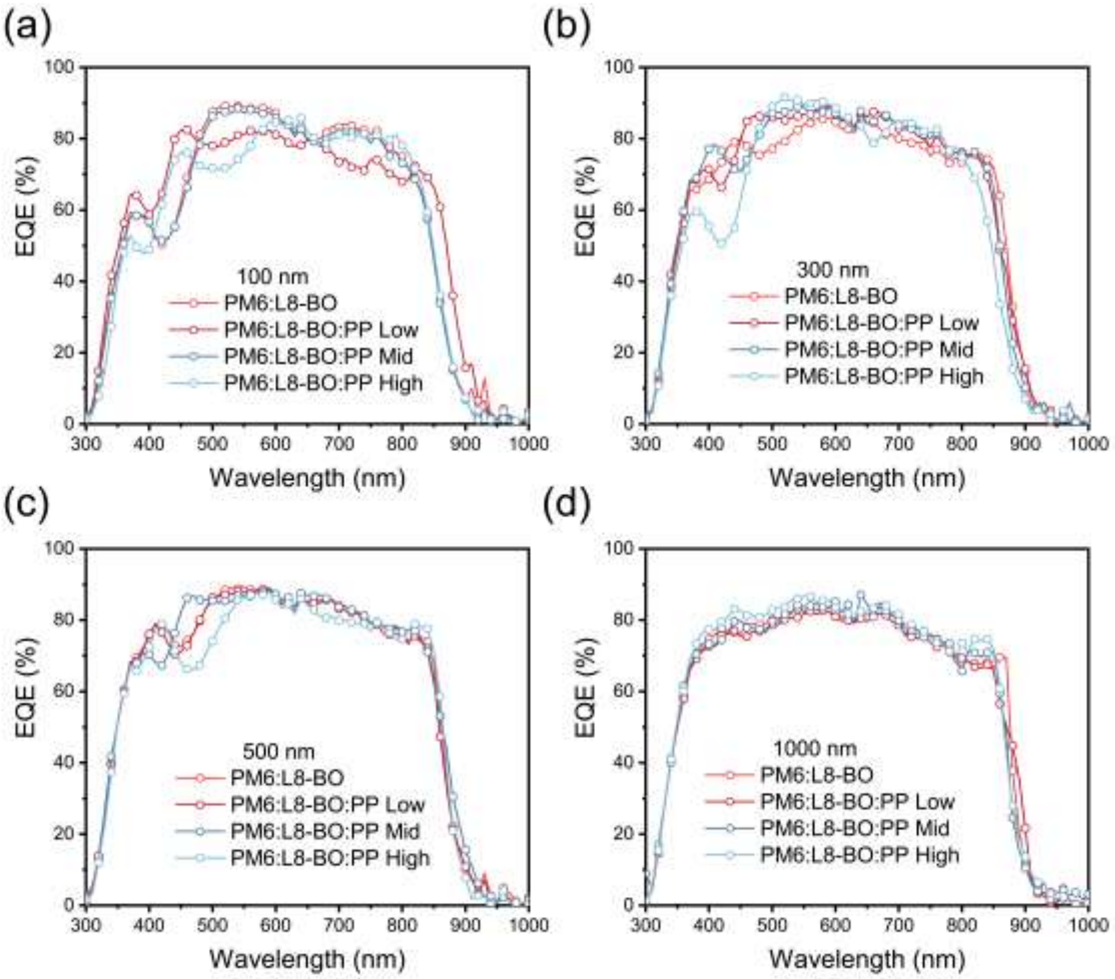

Figure S42. EQE spectra of the OSCs of (a) 100 nm, (b) 300 nm, (c) 500 nm and (d) 1000 nm system with different conditions.

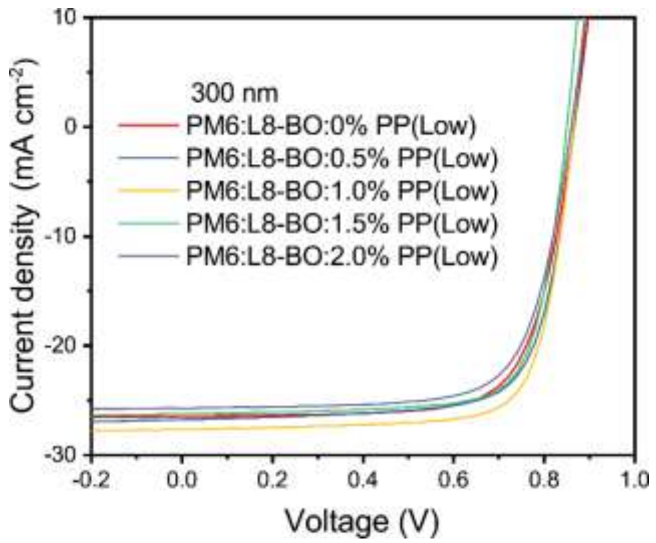

Figure 43. Current density versus bias voltage (*J*–*V*) curves of PM6:L8-BO with different PP ratio.

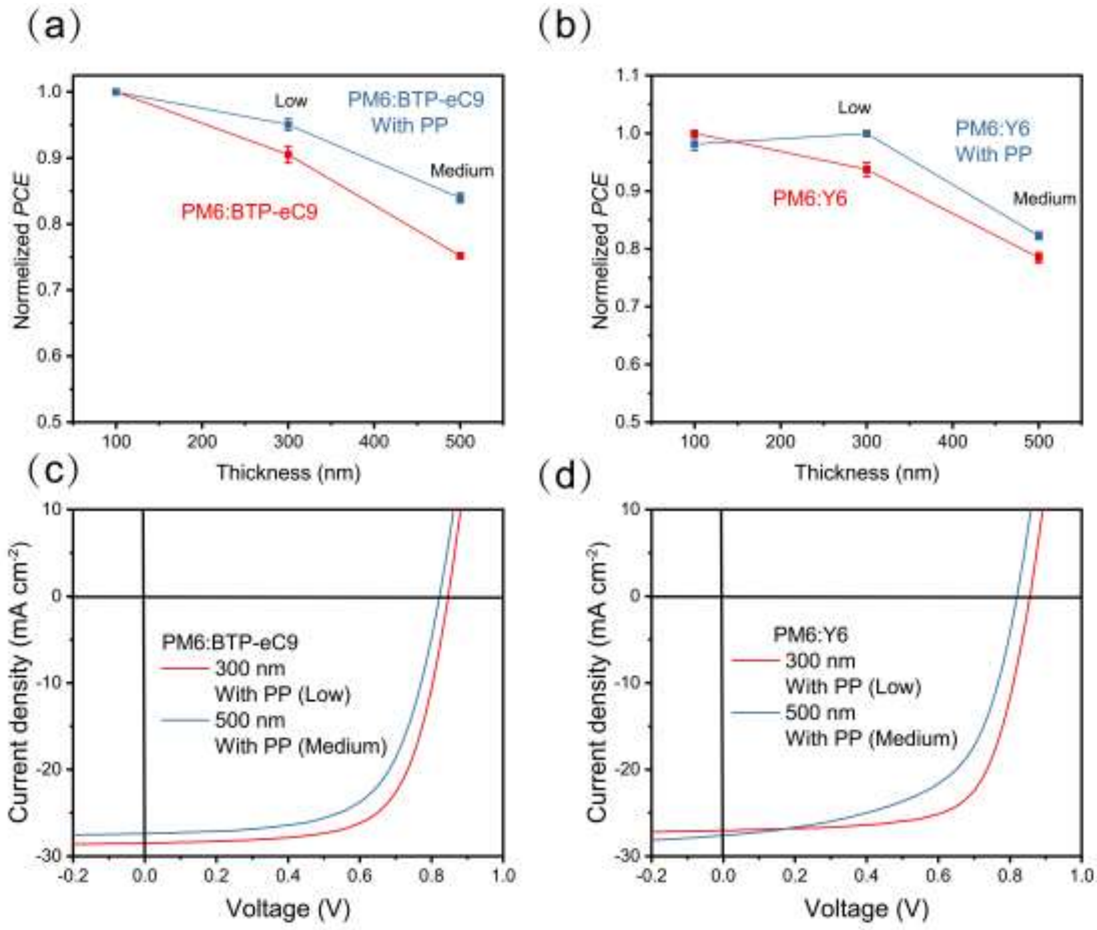

Figure S44. a) The variation trend of PCE increases with the active layer thickness. b) Current density versus bias voltage (J–V) curves of the OSCs of different systems.

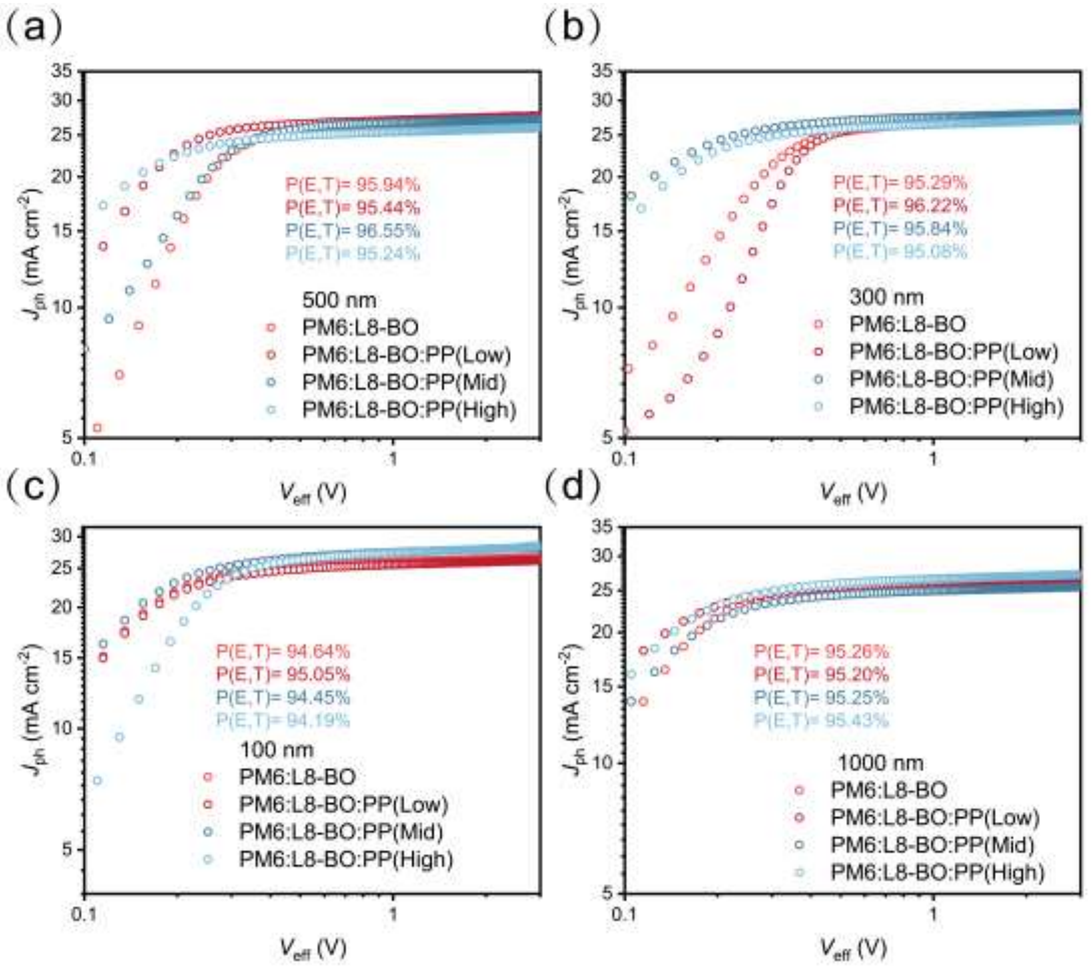

Figure S45. The evolution of J_{ph} versus V_{eff} for the different thickness devices.

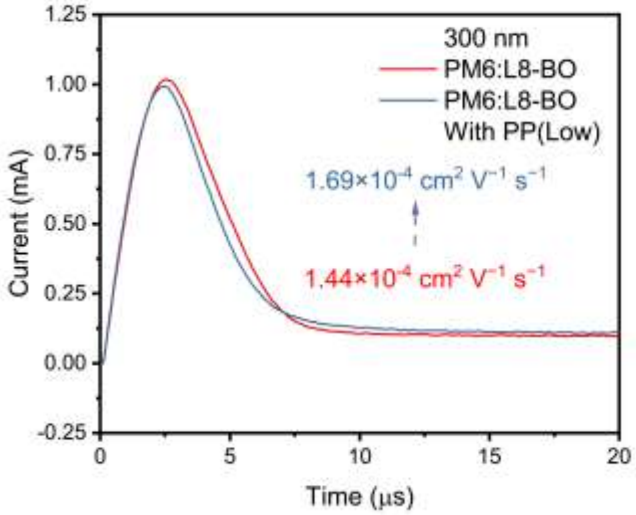

Figure S46. Photo-CELIV curves of the 300 nm devices with different thickness.

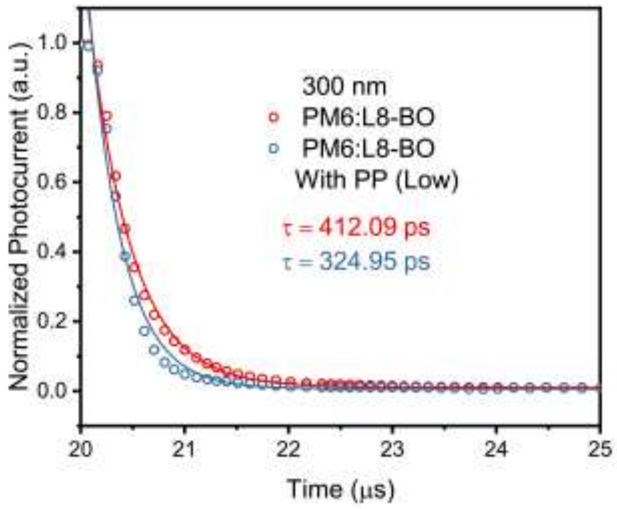

Figure S47. Normalized TPC data for the 300 nm devices with different thickness.

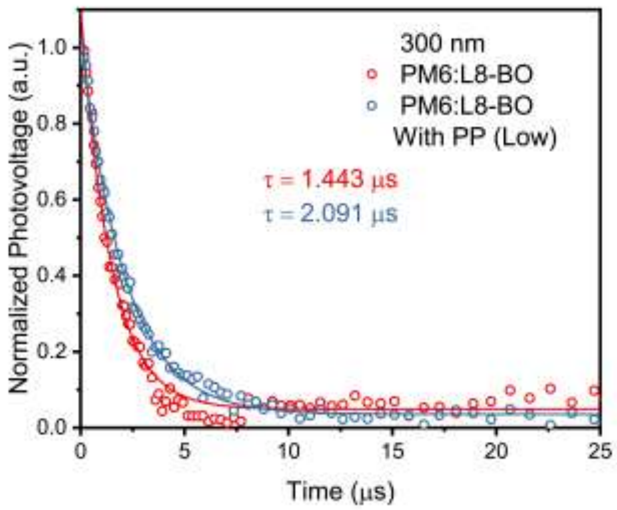

Figure S48. Normalized TPV data for the 300 nm devices with different thickness.

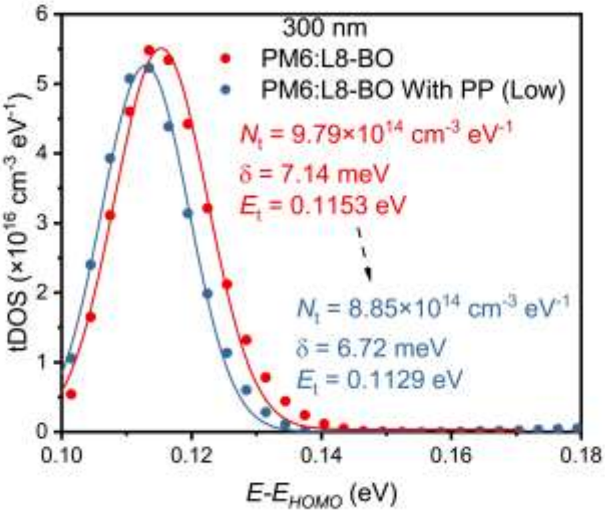

Figure S49. DoSs of 300 nm blended films and corresponding Gaussian fitting results.

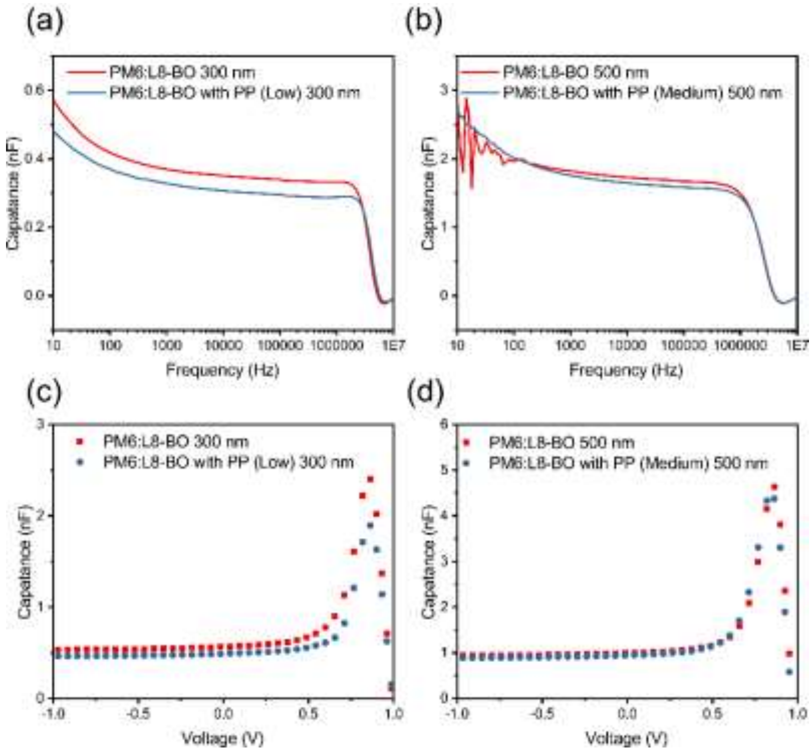

Figure S50. (a)-(b) Capacitance-Frequency spectra of the corresponding devices. (c)-(d). Capacitance-Voltage spectra of the corresponding devices.

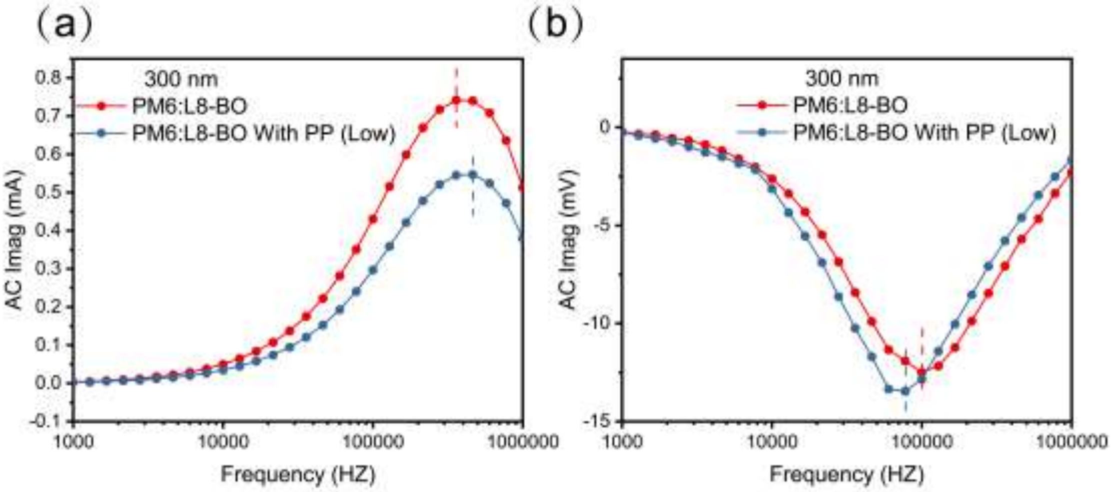

Figure S51. IMVS (a) and IMPS (b) curves of 300 nm devices with different thickness.

Table S1. Summary of photovoltaic parameters under AM 1.5 G.

System	Thickness (nm)	J_{SC} [mA/cm^2][b)]	V_{OC} [V][b)]	FF [%][b)]	PCE [%][b)]
PM6:Y6	100	25.16 (25.04±0.14)	0.856 (0.856±0.001)	72.41 (72.04±0.38)	15.60 (15.44±0.13)
	200	25.48 (25.23±0.28)	0.832 (0.832±0.003)	71.34 (71.26±0.23)	15.13 (14.97±0.15)
	300	26.56 (26.35±0.24)	0.830 (0.829±0.001)	66.33 (66.21±0.39)	14.62 (14.47±0.13)
	400	26.86 (26.60±0.23)	0.815 (0.815±0.001)	57.90 (57.72±0.30)	12.68 (12.52±0.10)
	500	26.21 (26.04±0.21)	0.818 (0.818±0.001)	57.04 (56.89±0.25)	12.22 (12.11±0.09)

[a)] $J_{cal.}$ represents the integrated current density obtained from EQE spectra;

[b)] Average values with standard deviation were obtained from 10 individual device.

Table S2. Summary of photovoltaic parameters under AM 1.5 G.

System	Thickness (nm)	J_{SC} [mA/cm^2][b)]	J_{CAL} [mA/cm^2] [a)]	V_{OC} [V][b)]	FF [%][b)]	PCE [%][b)]
PM6:L8-BO	100	25.72 (25.51±0.25)	24.89	0.898 (0.899±0.001)	78.69 (78.34±0.33)	18.17 (17.96±0.20)
	200	26.30 (26.20±0.13)	25.15	0.874 (0.873±0.002)	75.60 (75.10±0.44)	17.37 (17.18±0.13)
	300	26.46 (26.30±0.17)	25.28	0.866 (0.865±0.001)	72.39 (71.84±0.56)	16.58 (16.35±0.18)
	400	26.62 (26.31±0.40)	25.56	0.863 (0.862±0.002)	67.29 (67.29±0.42)	15.45 (15.25±0.18)
	500	27.09 (26.86±0.30)	25.59	0.843 (0.844±0.001)	61.88 (61.44±0.51)	14.13 (13.92±0.19)

[a)] $J_{cal.}$ represents the integrated current density obtained from EQE spectra;

[b)]Average values with standard deviation were obtained from 8 individual device.

Table S3. Summary of photovoltaic parameters under AM 1.5 G.

System	Thickness (nm)	J_{SC} [mA/cm^2][b)]	J_{CAL} [mA/cm^2][a)]	V_{OC} [V][b)]	FF [%][b)]	PCE [%][b)]
PM6:BTP-eC9	100	26.55 (26.35±0.21)	25.70	0.845 (0.844±0.002)	76.76 (76.31±0.46)	17.21 (16.97±0.18)
	200	26.85 (26.37±0.39)	26.05	0.827 (0.827±0.001)	71.61 (71.50±0.47)	15.90 (15.59±0.22)
	300	27.63 (27.36±0.31)	26.95	0.832 (0.832±0.002)	67.59 (67.49±0.25)	15.53 (15.36±0.16)
	400	27.26 (27.03±0.31)	26.56	0.820 (0.821±0.003)	66.13 (65.86±0.44)	14.79 (14.61±0.14)
	500	27.20 (26.98±0.25)	26.46	0.817 (0.816±0.002)	65.26 (65.11±0.47)	14.50 (14.33±0.14)

[a)]$J_{cal.}$ represents the integrated current density obtained from EQE spectra;

[b)]Average values with standard deviation were obtained from 8 individual device.

Table S4. Key parameters of contact angle measurements by using water and glycerol droplets.

Materials	Contact angle [°]		Surface Energy γ[mNm^{-1}]	Systems	Flory-Huggins interaction parameter (χ)
	Water	Glycerin			
PM6 300 nm	107.58	87.41	22.92	PM6 300 nm with PP (Low)	0.54
PM6 500 nm	103.51	82.71	30.57	L8-BO 300 nm with PP (Low)	0.08
L8-BO 300 nm	102.88	84.56	33.45	PM6 500 nm with PP (Medium)	3.07
L8-BO 500 nm	96.57	75.67	43.24	L8-BO 500 nm with PP (Medium)	0.50
PP (Low)	74.75	67.23	30.51		
PP (Medium)	76.46	53.92	53.01		

Table S5. Fitting parameters of temperature-related Peak Location and FWHM of the PL peaks

System	$E(0)(meV)$	$a(meV)$	$\Theta(k)$	β(μeV K^{-1})	E_A (meV)
L8-BO 300 nm	1.323	121.23	422.13	380.90	439.51
L8-BO 300 nm with PP (Low)	1.325	109.35	408.16	347.78	312.97
L8-BO 500 nm	1.323	149.70	473.73	388.47	483.42
L8-BO 500 nm with PP (Medium)	1.32334	132.67	446.82	369.98	329.89

Table S6. Summary of GIWAXS π-π stacking information in the OOP direction of different systems.

	System	q [$Å^{-1}$]	d-spacing [Å]	FWMH [$Å^{-1}$]	CCL [Å]
300 nm	L8-BO	1.86	3.38	0.392	14.411
	L8-BO with PP (Low)	1.87	3.36	0.286	19.787
500 nm	L8-BO	1.86	3.38	0.385	14.702
	L8-BO with PP (Medium)	1.88	3.34	0.244	23.155

Table S7. Summary of GIWAXS π-π stacking information in the OOP direction of different systems.

	System	q [$Å^{-1}$]	d-spacing [Å]	FWMH [$Å^{-1}$]	CCL [Å]
300 nm	PM6:L8-BO	1.86	3.37	0.213	26.597
	PM6:L8-BO with PP (Low)	1.86	3.37	0.198	28.497
500 nm	PM6:L8-BO	1.87	3.36	0.197	28.765
	PM6:L8-BO with PP (Medium)	1.87	3.36	0.194	29.182

Table S8. Detailed Hole Transfer Parameters of different systems

Thickness (nm)	System	A_1 (%)	τ_1[ps]	A_2 (%)	τ_2[ps]	τ_{avg}[ps]
300	PM6:L8-BO	80.16	0.241	19.85	1.910	0.572
	PM6:L8-BO with PP (Low)	77.32	0.162	22.68	1.158	0.388
500	PM6:L8-BO	97.10	0.273	2.90	7.294	0.476
	PM6:L8-BO with PP (Medium)	92.92	0.199	7.08	1.440	0.287

Table S9. Fitting parameters for the pump fluence-dependent TA decay curve.

System	τ [ps]	k [ps^{-1}]	γ [$\times 10^{-8}$ cm^3 s^{-1}]	D [$\times 10^{-2}$ cm^2 s^{-1}]	L_D [nm]
L8-BO 500 nm	653.94	1.53	2.27	0.90	24.32
L8-BO 500 nm With PP (Medium)	665.52	1.50	3.25	1.29	29.35

Table S10. Summary of Static Disorder ($E_U(0)$), Dynamic Disorder ($E_{U,t}$), and Einstein Temperature θ_E.

	System	$E_U(0)$(meV)	$E_{U,t}$(meV)	θ_E(K)
300 nm	PM6:L8-BO	22.78	14.51	548.16
	PM6:L8-BO with PP (Low)	20.14	14.03	334.19
500nm	PM6:L8-BO	27.76	15.90	392.07
	PM6:L8-BO with PP (Medium)	24.24	14.80	367.39

Table S11. Summary of photovoltaic parameters under AM 1.5 G.

Thickness (nm)	System	J_{SC} [mA/cm^2][b)]	J_{CAL} [mA/cm^2][a)]	V_{OC} [V][b)]	FF [%][b)]	PCE [%][b)]
100	PM6:L8-BO	25.72 (25.51±0.25)	24.57	0.898 (0.897±0.001)	78.69 (78.34±0.33)	18.17 (17.96±0.20)
	PM6:L8-BO with PP (Low)	25.48 (25.36±0.14)	24.52	0.891 (0.892±0.001)	78.41 (78.15±0.25)	17.81 (17.69±0.12)
	PM6:L8-BO with PP (Medium)	25.26 (25.06±0.26)	24.15	0.881 (0.882±0.003)	77.42 (77.17±0.41)	17.24 (17.05±0.15)
	PM6:L8-BO with PP (High)	24.96 (24.67±0.25)	23.88	0.878 (0.878±0.001)	76.24 (76.23±0.19)	16.71 (16.51±0.18)
300	PM6:L8-BO	26.46 (26.30±0.17)	25.44	0.866 (0.867±0.001)	72.39 (71.84±0.56)	16.58 (16.35±0.18)
	PM6:L8-BO with PP (Low)	27.64 (27.51±0.20)	26.03	0.864 (0.864±0.002)	75.61 (75.47±0.20)	18.06 (17.93±0.11)
	PM6:L8-BO with PP (Medium)	27.03 (26.78±0.15)	25.92	0.865 (0.864±0.001)	70.23 (70.22±0.51)	16.41 (16.26±0.12)
	PM6:L8-BO with PP (High)	26.05 (25.89±0.17)	24.74	0.858 (0.859±0.001)	68.06 (67.91±0.28)	15.21 (15.10±0.08)
500	PM6:L8-BO	27.09 (26.86±0.30)	26.00	0.843 (0.844±0.001)	61.88 (61.44±0.51)	14.14 (13.92±0.20)
	PM6:L8-BO with PP (Low)	26.98 (26.74±0.30)	25.70	0.840 (0.840±0.001)	61.33 (61.21±0.23)	13.91 (13.75±0.15)
	PM6:L8-BO with PP (Medium)	27.64 (26.74±0.30)	26.23	0.853 (0.853±0.001)	67.48 (67.34±0.46)	15.92 (15.73±0.15)
	PM6:L8-BO with PP (High)	26.23 (26.12±0.17)	25.09	0.857 (0.857±0.002)	62.35 (62.29±0.11)	14.01 (13.95±0.08)
1000	PM6:L8-BO	26.36 (26.23±0.14)	25.24	0.841 (0.841±0.001)	48.87 (48.67±0.52)	10.83 (10.73±0.09)
	PM6:L8-BO with PP (Low)	26.13 (25.94±0.26)	24.83	0.846 (0.845±0.002)	48.57 (48.34±0.49)	10.73 (10.60±0.10)
	PM6:L8-BO with PP (Medium)	26.29 (26.23±0.16)	25.12	0.843 (0.843±0.002)	48.71 (49.50±0.31)	10.79 (10.71±0.06)
	PM6:L8-BO with PP (High)	26.94 (26.78±0.18)	25.65	0.852 (0.852±0.002)	51.90 (51.78±0.29)	11.92 (11.82±0.09)

[a)] $J_{cal.}$ represents the integrated current density obtained from EQE spectra;

[b)] Average values with standard deviation were obtained from 8 individual device.

Table S12. Summary of photovoltaic parameters under AM 1.5 G.

System	J_{SC} [mA/cm^2]	V_{OC} [V]	FF [%]	PCE [%][a)]
PM6:L8-BO+0%PP	26.46	0.866	72.39	16.58(16.35±0.18)
PM6:L8-BO+0.5%PP	26.75	0.866	73.42	16.99(16.77±0.21)
PM6:L8-BO+1.0%PP	27.64	0.864	75.61	18.060(17.93±0.11)
PM6:L8-BO+1.5%PP	26.21	0.850	75.60	16.84(16.74±0.10)
PM6:L8-BO+2.0%PP	25.72	0.860	71.84	15.89(15.80±0.08)

[a)]Average values with standard deviation were obtained from10 individual device.

Table S13. Quantitative calculation parameters for IMPS and IMVS based on different devices.

Thickness (nm)	System	τ_{tr} (s)	τ_{rec} (s)	η (%)
300	PM6:L8-BO	4.43×10^{-7}	1.59×10^{-6}	72.17
	PM6:L8-BO with PP (Low)	3.43×10^{-7}	2.06×10^{-6}	83.32
500	PM6:L8-BO	7.39×10^{-7}	2.06×10^{-6}	64.62
	PM6:L8-BO with PP (Medium)	5.72×10^{-7}	2.65×10^{-6}	78.46

References

[1] T. Wang, M.-S. Niu, J.-J. Guo, K.-N. Zhang, Z.-C. Wen, J.-Q. Liu, C.-C. Qin, X.-T. Hao, *Solar RRL* **2020**, 4.

[2] S. Kouijzer, J. J. Michels, M. van den Berg, V. S. Gevaerts, M. Turbiez, M. M. Wienk, R. A. Janssen, *J Am Chem Soc* **2013**, 135, 12057.

[3] S. Liu, Y. Jing, J. Tu, H. Zou, Z. Yong, G. Liu, *J. Appl. Polym. Sci.* **2022**, 139.

[4] X. Jiang, X. Yuan, X. Guo, F. Zeng, H. Wang, G. Liu, *Fluid Phase Equilib.* **2023**, 563.

[5] Y. Xiao, X. Lu, *Materials Today Nano* **2019**, 5.

[6] F. Cui, J. Qiao, Y. Xu, Z. Fu, R. Gui, C. Zhang, R. Zhou, L. Ye, X. Du, F. Chen, X. Hao, H. Yan, H. Yin, *Sci Adv* **2024**, 10, eado5460.

[7] L. Zhu, M. Zhang, J. Xu, C. Li, J. Yan, G. Zhou, W. Zhong, T. Hao, J. Song, X. Xue, Z. Zhou, R. Zeng, H. Zhu, C.-C. Chen, R. C. I. MacKenzie, Y. Zou, J. Nelson, Y. Zhang, Y. Sun, F. Liu, *Nat. Mater.* **2022**, 21, 656.

[8] G. Zhou, M. Zhang, Z. Chen, J. Zhang, L. Zhan, S. Li, L. Zhu, Z. Wang, X. Zhu, H. Chen, L. Wang, F. Liu, H. Zhu, *ACS Energy Lett.* **2021**, 6, 2971.

[9] Z. H. Chen, H. F. Yao, J. W. Wang, J. Q. Zhang, T. Zhang, Z. Li, J. W. Qiao, S. S. Xiu, X. T. Hao, J. H. Hou, *Energy Environ. Sci.* **2023**, 16, 2637.
[10] L. Bu, S. Gao, W. Wang, L. Zhou, S. Feng, X. Chen, D. Yu, S. Li, G. Lu, *Advanced Electronic Materials* **2016**, 2.
[11] X. Li, F. Pan, C. Sun, M. Zhang, Z. Wang, J. Du, J. Wang, M. Xiao, L. Xue, Z.-G. Zhang, C. Zhang, F. Liu, Y. Li, *Nat. Commun.* **2019**, 10, 519.
[12] C. Han, B. Cheng, Q. Guo, Z. Fu, J. Qiao, S. Cheng, Y. Huo, X. Xia, H. Wang, Y. Fu, X. Guo, X. Lu, X. Hao, Y. Li, M. Zhang, *Adv. Funct. Mater.* **2024**, 2416381.
[13] C. Lee, W. Yang, R. G. Parr, *Phys. Rev. B* **1988**, 37, 785.
[14] R. Krishnan, J. S. Binkley, R. Seeger, J. A. Pople, *J. Chem. Phys.* **1980**, 72, 650.
[15] S. Grimme, S. Ehrlich, L. Goerigk, *J. Comput. Chem.* **2011**, 32, 1456.
[16] C. I. Bayly, P. Cieplak, W. Cornell, P. A. Kollman, *The Journal of Physical Chemistry* **2002**, 97, 10269.
[17] T. Lu, F. Chen, *J. Comput. Chem.* **2012**, 33, 580.
[18] J. Wang, R. M. Wolf, J. W. Caldwell, P. A. Kollman, D. A. Case, *J. Comput. Chem.* **2004**, 25, 1157.
[19] A. W. Sousa da Silva, W. F. Vranken, *BMC Res. Notes* **2012**, 5, 367.
[20] M. J. Abraham, T. Murtola, R. Schulz, S. Páll, J. C. Smith, B. Hess, E. Lindahl, *SoftwareX* **2015**, 1-2, 19.
[21] U. Essmann, L. Perera, M. L. Berkowitz, T. Darden, H. Lee, L. G. Pedersen, *J. Chem. Phys.* **1995**, 103, 8577.
[22] P. J. Steinbach, B. R. Brooks, *J. Comput. Chem.* **2004**, 15, 667.
[23] B. Hess, H. Bekker, H. J. C. Berendsen, J. G. E. M. Fraaije, *J. Comput. Chem.* **1997**, 18, 1463.
[24] R. W. Hockney, S. P. Goel, J. W. Eastwood, *J. Comput. Phys.* **1974**, 14, 148.
[25] G. Bussi, D. Donadio, M. Parrinello, *J. Chem. Phys.* **2007**, 126, 014101.
[26] M. Bernetti, G. Bussi, *J. Chem. Phys.* **2020**, 153, 114107.
[27] W. Humphrey, A. Dalke, K. Schulten, *J. Mol. Graph.* **1996**, 14, 33.
